# Composite Biopolymer-Based Wafer Dressings Loaded with Microbial Biosurfactants for Potential Application in Chronic Wounds

**DOI:** 10.3390/polym10080918

**Published:** 2018-08-15

**Authors:** Olufunke Akiyode, Joshua Boateng

**Affiliations:** Department of Pharmaceutical, Chemical and Environmental Sciences, Faculty of Engineering and Science, University of Greenwich, Kent ME4 4TB, UK; olufunkeakiyode@gmail.com

**Keywords:** biosurfactants, carrageenan, polymer composites, rhamnolipids, sodium alginate, sophorolipids, wafers, wound healing

## Abstract

In this study two bioactive polysaccharide polymers *kappa*-carrageenan (CARR) and sodium alginate (SA) incorporated with microbial biosurfactants (BSs) were formulated as medicated wafer dressings for potential application in chronic wounds. Wafers were loaded with BSs at concentrations of 0.1% and 0.2% rhamnolipids (RL) and 0.1% and 5% sophorolipids (SL) and were functionally characterized using scanning electron microscopy (SEM), texture analysis (mechanical strength and in vitro wound adhesion), attenuated total reflectance Fourier transform infrared (ATR-FTIR) spectroscopy, X-ray diffraction (XRD) and exudate handling properties (pore analysis, swelling index, water absorption (*A*w), equilibrium water content (*EWC*), evaporative water loss (EWL) and water vapor transmission rate (WVTR). The wafers were tactile and ductile in appearance with a hardness range of 2.7–4.1 N and can withstand normal stresses but are also flexible to prevent damage to newly formed skin tissues. Wafers were porous (SEM) with pore sizes ranging from 78.8 to 141 µm, and BSs were not visible on the wafer surface or pore walls. The BSs enhanced the porosity of the wafers with values above 98%, while the *A*w and *EWC* ranged from 2699–3569% and 96.58–98.00%, respectively. The EWL ranged from 85 to 86% after 24 h while the WVTR ranged from 2702–3080 g/m^2^ day^−1^. The compatibility of BSs within the CARR-SA matrix was confirmed by seven characteristic functional groups which were consistently transmitted in the ATR-FTIR spectra. These novel medicated dressing prototypes can potentially help to achieve more rapid wound healing.

## 1. Introduction

A wound could be described as a break in the continuity of the epithelial membrane of the skin or mucosa and may be attributed to physical or thermal damage or other factors such as disease, for example, diabetes [1]. Wound healing forms part of the process of tissue regeneration and progresses through four distinct but overlapping phases, which are hemostasis and inflammation, migration, proliferation and maturation phases [2,3]. Wounds can be classified as either acute or chronic, with the latter being characterized by failure to heal within 12 weeks as a result of continuous or extended inflammatory phase [1]. Chronic wounds arise because of repeated tissue trauma or underlying physiological conditions [4] including diabetes and cancers, uncontrolled infections, and poor clinical therapy combined with patient related factors such as lack of exercise and poor nutrition.

Chronic wounds include diabetic foot ulcers, decubitis ulcers (bedsores or pressure sores) and leg ulcers (venous, ischemic or of traumatic origin) [5]. During injury, various foreign objects could be embedded within the resulting wound and this can result in chronic inflammatory responses which delay tissue regeneration and may subsequently cause granuloma or the formation of abscesses. Improper management of wound infections can lead to inflammation and this may result in bacteremia and septicemia, which could result in fatalities. Geriatric patients and those on a poor diet also have reduced ability to fight infection [6]. Further, protein, vitamin (e.g., vitamin C) and mineral deficiencies can impact negatively on collagen synthesis and the inflammatory phase [7]. In addition, chronic wounds generally produce significant quantities of exudate, resulting in patients having to wear soiled and heavy dressings that regularly leak and have foul odor, whilst surrounding healthy skin could get macerated due to excessive collection of exudate underneath dressings. Heavy exudate production therefore has both economic (soiling and regular laundering) and social (embarrassment, social isolation and mental health) implications [8]. The ideal wound dressing is therefore expected to reduce and control pain, prevent excessive loss of fluid from the wound site whilst maintaining a moist environment. It must enhance restoration of the epithelial tissue and should not be toxic to newly formed skin cells (that is biocompatible). In addition, the dressing should be bioadhesive and adhere readily to the wound whilst being easy to remove without causing trauma and bleeding and pain to the patient [1] which is important for ensuring patient comfort and compliance. Further, repeated changing of dressings by either the healthcare provider or the patient continues until the wound heals but this is often an inconvenient process and needs some basic improvement [9].

Wafers are obtained by freeze-drying of polymeric solutions to produce solid porous matrices that can easily be applied to exuding wound surfaces [10,11]. Furthermore, wafers have been reported to possess better drug-loading capacity compared to their corresponding films [12]. The physical stability of a freeze-dried wafer preserves the size, shape and physical form of incorporated drugs unlike formulations such as gels, which are prone to undesirable processes such as crystal ripening, agglomeration and polymorphism [13]. In addition, the high porosity of wafers increase their ability to imbibe exudate and swell, whilst maintaining their structural gel integrity. This allows the maintenance of a moist wound healing environment without damaging newly formed tissue [14].

K-carrageenan (CARR) and sodium alginate (SA) are naturally derived polysaccharide polymers selected for the formulation of composite wafers due to their known biocompatibility (non-toxicity), ease of biodegradation, and low immunogenicity [15]. Various authors have reported on composite formulations containing one or both of these two polymers. Roh and Shin [16], showed that the pore size of CARR and SA composite formulations decreased as the concentration of CARR increased as well as when crosslinked in the presence of calcium chloride. Popa and co-workers [17] observed that the pore size of composite CARR and SA hydrogels were homogenously distributed but increased with increasing CARR content greater than 60%. In a study by Pascalau and co-workers [18], it was shown that composite formulations combining CARR and SA exhibited improved mechanical characteristics and swelling behavior when compared to those obtained from CARR and SA alone. Pawar and co-workers [19] reported composite blends of polyox with CARR and polyox with SA with excellent swelling and adhesion profiles for simultaneous delivery of streptomycin and diclofenac to target both infection and pain of inflammation associated with chronic wounds.

Unlike traditional dressings such as gauze and cotton wool that take no active part in the wound healing process, advanced medicated dressings are designed to have biological activity either on its own or the release of bioactive constituents (drugs) incorporated within the dressing [2,3]. Medicated polymeric (synthetic, semi-synthetic or naturally derived) dressings are potentially useful in the treatment of local infections where it may be beneficial to achieve increased local concentrations of antibiotics while avoiding high systemic doses, thus reducing patient exposure to an excess of drug beyond that required at the wound site [20]. The inflammatory phase of wound healing is the main problem in chronic wounds, and BSs are reported to have anti-inflammatory activities [21], and therefore potential wound healing effect. Biosurfactants (BSs) are broad-spectrum compounds with reported therapeutic activity including anti-microbial and anti-inflammatory effects, making them potential targets for chronic wound healing [21]. Rhamnolipids (RL) and sophorolipids (SL) are glycolipid-based BSs produced by microorganisms such as bacteria and yeast. Rhamnolipids belong to a family of congeners differentiated by the substituent mono and di-rhamnose sugars that can be attached to one or two hydrocarbon chains, respectively. RL possess anti-bacterial activity against a number of organisms including *Listeria monocytogenes* [22], *Pseudomonas aeruginosa* and *Bacillus subtilis* [23] and can therefore be loaded within hydrogels or as dry soluble solids [24]. RL have been reported for use in enhancing the re-epithelization of mucous membranes including treating gum disease and for periodontal regeneration [25]. In addition, low concentrations of RL can inhibit the phagocytic actions of macrophages [26] which is important for controlling the inflammatory phase. Stipceviv and colleagues [25] formulated ointments loaded with di-rhamnolipid BAC-3 for topical application on full thickness burn wounds in normal Sprague-Dawley rats covering 5% of the total body surface area and showed improvements in healing. They exist in two main forms: lactonic or acidic and can be incorporated in hydrogels either as solutions of dissolved molecules, ions or as dry soluble (acidic), insoluble (lactonic) or as a soluble intermediate mixture of both forms [24]. SL have been used in skin moisturizers, possess mild antibacterial activity [27,28] and improve skin cell growth by stimulating fibroblast metabolism and collagen synthesis [29]. Other biological activities include anticancer [30,31], and anti-inflammatory effects [32,33].

This study involves the incorporation of BSs into lyophilized wafer wound dressings comprising a composite mixture of k-CARR and SA. Although Ju et al. [24] patented the mixture of RL and SL in gelatin-alginate hydrogel wound dressings, to the best of our knowledge this is the first comprehensive formulation development and characterization of RL- and SL-loaded composite (CARR:SA based) freeze-dried wafers. The possible synergistic effects of combining two natural biomaterial polymers on the mechanical properties and fluid handling properties of the resulting wafers are proposed. The hypothesis is that the incorporation of microbial based BSs within a composite biopolymer matrix comprising CARR and SA will exhibit functional characteristics expected of an advanced moist dressing for potential application in chronic (hard-to-heal) wounds. The effects of total polymer weight and/or ratio of CARR:SA have been studied by physico-chemical characterization which allowed the selection of optimized formulations for BSs loading. Physico-chemical and analytical techniques used include scanning electron microscopy, texture analysis (mechanical strength and adhesion), ATR-FTIR, XRD and exudate handling properties.

## 2. Materials and Methods

### 2.1. Materials

Rhamnolipid (R-90™), sodium phosphate tribasic, dodecahydrate (*>*98%), bovine serum albumin (BSA) were all purchased from Sigma-Aldrich (Gillingham, UK), while sophorolipid (REWOFERM SL ONE) was kindly donated by Evonik Nutrition & Care GmbH Golschmidstr, Essen, Germany. Kappa carrageenan–CARR with high sulfation levels (Gelcarin GP 812 NF) was obtained from IMCD Ltd. (Sutton, UK); Sodium alginate (SA) (MW range 120,000–190,000 g/mol and ratio of mannuronic acid to guluronic acid = 1.56), sodium chloride, tris(hydroxy)aminomethane, calcium chloride dehydrate and ethanol (laboratory grade) were all purchased from Fisher Scientific (Loughborough, UK). Gelatin was obtained from Fluka Analytical (Steinheim, Germany) and calcium chloride from Sigma-Aldrich (Steinheim, Germany).

### 2.2. Methods

Blank (BLK) CARR and SA gels at various concentrations ranging from 1.0 to 3.0% *w*/*w* were initially formulated. This was followed by further formulation development and optimization of BLK composite (CARR and SA) gels at concentrations of (1.0–2.0% *w*/*w* total polymer) with various CARR:SA ratios; 1:1, 1:2, 1:3, 2:1 and 3:1. All gels were prepared by stirring on a magnetic stirrer at 70–90 °C. The drug-loaded (DL) gels were prepared by the addition of BSs to the selected optimized composite CARR: SA (1:3) polymeric gel (1.5% *w*/*w*) to obtain final BSs concentrations of 0.1% and 0.2% RL, 0.1%, 0.2% and 5% SL, as summarized in Table 1. The homogeneous gels (1 g) were poured into each mold of twenty four-well plates (diameter 35 mm) (CorningR_CellBINDR; Sigma Aldrich) and then freeze-dried in a Virtis Advantage XL 70 freeze dryer (Biopharma Process Systems, Winchester, UK) using an established cycle. Briefly, samples were cooled to −5 °C from room temperature for 1 h, and further frozen at −50 °C for 8 h (at 200 mTorr). The frozen samples were then primary dried to −25 °C (at 50 mTorr) in a sequence of thermal ramps for 24 h, followed by secondary drying at 20 °C (at 10 mTorr) for 7 h [19]. Lyophilized wafers were then removed from the freeze-dryer and stored in glass desiccators containing silica gel until required for characterization.

### 2.3. Scanning Electron Microscopy

Surface morphology of the lyophilized wafers was analyzed by a Hitachi SU 8030 (Hitachi High-Technologies, Krefeld, Germany) scanning electron microscope at an accelerating voltage of 1 kV. Wafers were cut into thin slices and mounted on aluminium stubs (15 mm diameter) with “Agar Scientific G3347N” double-sided-adhesive carbon tabs and splutter coated with chromium at 125 mA for 80 s. Images of the wafers were acquired at a working distance of approximately 15.0 mm at magnifications of 500×–1500×.

### 2.4. X-ray Diffraction

X-ray diffraction analyses of the prepared wafers and starting materials were performed using a D8 Advance X-ray diffractometer (Bruker AXS GmbH, Karlsure, Germany). The wafers were compressed to 0.5 mm using a clean pair of compression glasses and mounted on the sample holder. The transmission diffractograms were acquired in the range of 5° to 60° 2-theta, step size of 0.02° at a scan speed of 0.4 s per step. Diffraction patterns were obtained with DIFFRAC plus XRD commander software (Bruker AXS GmbH, Karlsure, Germany). The copper X-ray tube was set to 40 kV and 40 mA. A Göbel mirror was used which produced a parallel beam of CuKα radiation (*λ* = 1.54184 Å) using a divergent slit of 0.6 mm. A Lynx Eye position sensitive detector was used with an opening of 3°.

### 2.5. Attenuated Total Reflectance Fourier Transform Infrared Spectroscopy (ATR-FTIR)

An FTIR spectrophotometer was used in combination with (Thermo Nicolet; Thermo Scientific, Loughborough, UK), ZnSe attenuated total reflectance (ATR) accessory to characterize the wafers. The FTIR was equipped with potassium bromide (KBr) beam splitter and MCT detector. The wafers were placed on ZnSe ATR crystal and maximum pressure was applied using a pressure clamp accessory to allow for intimate contact of the wafers with the ATR crystal. Similarly, the pure starting materials (CARR, SA, RL and SL) were analyzed as controls. Spectra were recorded at 4 cm^−1^ resolution within a range of 400–4000 cm^−1^ using OMNICR_ software. True absorbance of each sample was obtained by background subtracting spectral information for the ATR crystal.

### 2.6. Mechanical Strength (‘Hardness’)

The mechanical properties (resistance to compressive deformation) of the freeze-dried wafers were investigated on a Texture Analyzer (Stable Microsystems Ltd., Surrey, UK) equipped with a 5 kg load cell and *Texture Exponent-32R* software program. All BLK and final optimized DL wafers were compressed using a 6 mm (P/6) cylindrical stainless steel probe (Stable Microsystems Ltd.) in compression mode. The effects of total polymer content and different combinations of CARR:SA were evaluated. The ‘hardness’ (resistance to deformation) of the wafers was evaluated by compressing the samples at five different locations (*n* = 4) to a depth of 1 mm at a speed of 0.20 mm/s using a trigger force of 0.001 N and withdrawn until it lost complete contact with the wafer. The same settings were used to determine the effects of BSs concentrations in the gels (0.1–5%, *w*/*w*) on optimized composite wafers [1.5% CARR:SA (1:3)].

### 2.7. Fluid Handling Properties

#### 2.7.1. Swelling Studies

The swelling studies were carried out as previously described [19]. In brief, the wafers were immersed in simulated wound fluid (SWF) containing (2% BSA, 0.02 M calcium chloride, 0.4 M sodium chloride, 0.08 M tris(hydroxyl) aminomethane in deionized water, pH 7.5 adjusted using dilute HCl) at room temperature. The change in weight of the hydrated wafers began after the first 5 min, followed by a 10 min reading. Subsequent measurements were then determined every 15 min up to 240 min. The hydrated wafers and measuring cups were carefully blotted with tissue paper to remove excess SWF on the surfaces and then weighed immediately on an electronic balance (European Instruments, Oxford, UK). The effect of polymer and BSs on swelling performance was evaluated for the different formulations. Percentage swelling index *I*_s_ (%) was calculated using Equation (1) [19].

(1) $$I_{s}\;\left(\%\right)=\frac{W_{s}-W_{i}}{W_{i}}\times 100\;$$

where *W_i_* is the dry weight of samples before hydration and *W*_s_ is the swollen weight of samples at different times of hydration.

#### 2.7.2. Pore Analysis

The porosity of wafers was determined by the solvent displacement method as previously described [34]. The geometrical dimensions (thickness and diameter) of samples were measured by a digital Vernier caliper and total pore volume (*V*_o_) was calculated. After that, samples were weighed (*W*_o_) before immersing in 10 mL of absolute ethanol for 3 h to reach saturation. Ethanol displaced the void spaces within the wafers. Finally, the wafer samples were carefully removed from the solvent, quickly blotted with tissue paper to remove excess solvent and immediately weighed (*W*_t_) to avoid loss of ethanol because of its known volatility. The porosity of the dressings was calculated from Equation (2) [35].

(2) $$Porosity\;\left(\%\right)=\;\frac{W_{t}-W_{o}\;}{\rho_{eth}V_{o}}\times 100\;$$

*ρ*_eth_: density of ethanol = 0.789 g/cm^3^.

#### 2.7.3. Water Absorption (*A*w) and Equilibrium Water Content (*EWC*)

Water absorption (*A*w) and equilibrium water content (*EWC*) tests were performed to investigate the maximum water uptake and water holding capacities, respectively, of BLK and BSs loaded wafers. Wafers were incubated in 5 mL of SWF at 37 °C continuously for 24 h. Before weighing, the samples were blotted carefully with tissue paper to remove excess fluid on the surface. The effect of drug (BSs) concentration in these studies was determined and the experiments were performed in triplicate (*n* = 3) for each sample. Percentage of (*A*w) and (*EWC*) were calculated by Equations (3) and (4) [36].

(3) $$\;Aw\;\left(\%\right)=\frac{W_{s}-W_{i}}{W_{i}}\times 100\;$$

(4) $$\;EWC\;\left(\%\right)=\frac{W_{s}-W_{i}}{W_{s}}\times 100\;$$

where *W*_s_ and *W_i_* are the swollen and initial weights before immersion into SWF, respectively.

#### 2.7.4. Evaporative Water Loss (*EWL*)

The SWF were drained from the same lyophilized wafer samples that had been incubated for *A*w and *EWC* after 24 h, measured and dried in the oven at 37 °C for another 24 h. The weight of the dried samples was recorded hourly for 6 h with a final reading after 24 h. Evaporative water loss (*EWL*) was calculated according to Equation (5) [36]:

(5) $$\;EWL\;\left(\%\right)=\;\frac{W_{t}}{W_{i}}\times 100\;$$

where *W*_t_ and *W*_i_ are the weight after time ‘*t’* and weight after initial 24 h immersion time, respectively.

#### 2.7.5. Water Vapor Transmission Rate (*WVTR*)

The wafers were mounted on the opening of a Falcon tube containing 4 mL water with 8 mm air gap between the samples and water surface. The whole set up was placed in an air-circulated oven at 37 °C for 24 h. The *WVTR* was calculated using Equation (6) [36]:

(6) $$WVTR=\frac{W_{i}-W_{t}}{A}\times 10^{6}\;g/m^{2}\;{\mathrm{day}}^{-1}$$

where *A* is the area of the opening of the Eppendorf tube (πr^2^), *W*_i_ and *W*_t_ are the weights of the whole set up before and after being placed into the oven, respectively.

### 2.8. Thermogravimetric Analysis (TGA)

The residual moisture content of the wafers was determined by thermogravimetric analysis (TGA) using a Q5000-IR TGA instrument (TA Instruments, Crawley, UK). About 1.0–1.5 mg of sample was loaded and analyzed with dynamic heating from room temperature (~25 °C) to 300 °C at a heating rate of 10 °C/min under inert nitrogen (N_2_) gas at a flow rate of 50 mL/min. The percentage water loss was calculated at 175 °C using *TA Instruments Universal Analysis 2000* software program.

### 2.9. In Vitro Adhesion Studies

Adhesive measurements were performed on the wafers using a TA.HD *plus* Texture Analyzer (Stable Microsystems Ltd.) fitted with a 5 kg load cell in tensile mode. The wafer (*n* = 4) was attached to an adhesive probe (75 mm diameter) using double-sided adhesive tape. The surface of a 6.67% (*w*/*v*) gelatin solution, allowed to set as a solid gel in a Petri dish (86 mm diameter), was equilibrated with 0.5 mL SWF containing 2% (*w*/*w*) BSA to mimic a wound surface. The probe, lined with wafer, was set to approach the model wound surface with a pre-test speed of 0.5 mm/s; test speed of 0.5 mm/s; post-test speed of 1.0 mm/s; applied force at 0.05 N; contact time of 60.0 s; auto trigger type; trigger force of 0.05 N and return distance of 10.0 mm. The adhesive characteristics were determined by the maximum force (stickiness) required to detach the wafer from the model wound surface; total work of adhesion (WOA) was represented by the area under the force versus distance curve, whereas cohesiveness was defined as the distance travelled by wafer till detached and calculated using the *Texture Exponent 32*R software (Stable Microsystems Ltd., Surrey, UK).

### 2.10. Statistical Analysis of Data

Data analysis was carried out with the Microsoft Excel version 2007 software package. Results were expressed as a mean ± standard deviation (S.D) of the respective replicates (*n* = 3 or 4). Statistical difference was determined using one-way analysis of variance (ANOVA) for the interrelation between the various groups with *p* < 0.05 considered as a minimal level of significance. The groups compared were the selected optimized BLK vs BSs loaded wafers and between the two different BSs loaded wafers.

## 3. Results and Discussion

### 3.1. Preliminary Formulation Development and Optimization

Preliminary formulation development initially involved using only CARR to produce gels for freeze-drying. However, formulation of wafers containing only CARR was discontinued due to rapid disintegration during swelling, implying CARR only wafers could not handle the significantly high exudate produced in chronic wounds. As a result, a composite mixture of CARR and SA was employed without impacting on the functional physical properties. Additionally, when combined, these two polymers, act synergistically as a result of the similarity in the type of polysaccharide gelling mechanism. CARR forms a gel with potassium and calcium ions but also shows gelation under salt-free conditions helped by physical bonds, and is a thermosensitive gel. Thermo-reversible gels, such as CARR, melt at elevated temperature and the gelation of the biopolymer is obtained by lowering the temperature. The temperature-induced gelation allows for an easy formation of gels with different shapes, due to the versatility of CARR. Compared to CARR, SA has been more extensively studied and characterized including as commercial moist wound dressings. Overall, the purpose of this work was to prepare medicated bioactive composite CARR:SA wafers loaded with BSs that could potentially be used as wound dressings. BLK wafers prepared from 1.5% (*w*/*w*) CARR:SA (1:3) gels from the preliminary studies showed desired characteristics required in wafers on the basis of an ideal balance between hardness (4–6 N), target porosity ≥65% [37] which corresponded to maximum water uptake and water holding capacities. This was therefore loaded with BSs and further characterized to compare with their corresponding BLK formulations. Other bio-based materials such as chitosan have been employed for similar applications to ensure enhanced mechanical properties. Siafaka and colleagues reported on porous chitosan-based dressings to deliver levofloxacin locally to the wound site [38]. However, though chitosan is an effective biomaterial for such applications, it is insoluble at high pH and therefore insoluble in water and requires acidification using acetic acid, which can be a limitation. Both CARR and SA are readily soluble in water as was the case in our study, which is a major advantage over use of chitosan.

### 3.2. Scanning Electron Microscopy (SEM)

**Figure 1:**
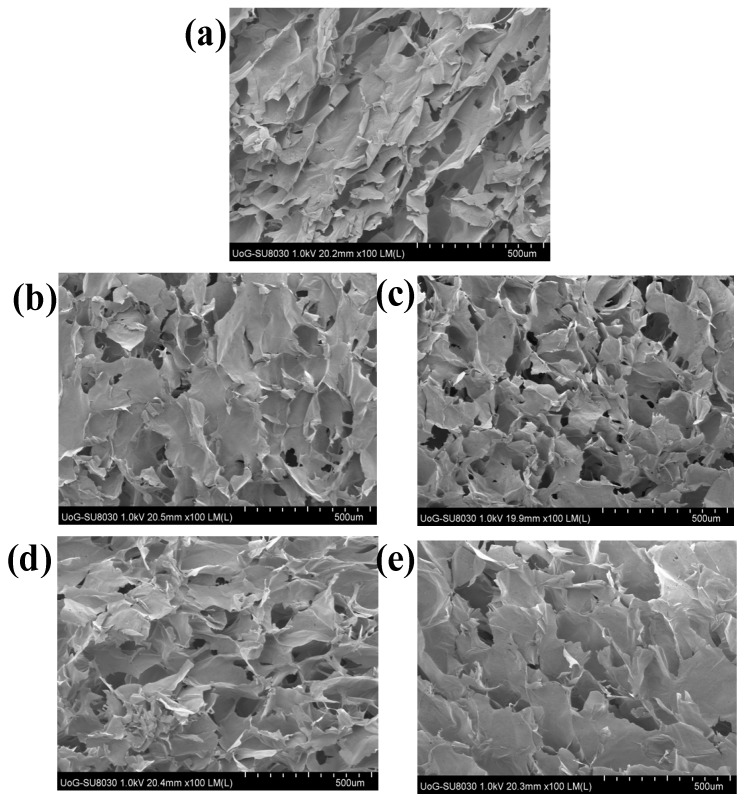
Scanning electron microscopy (SEM) images of (**a**) selected optimized BLK 1.5% CARR:SA (1:3), and their corresponding BSs loaded wafers i.e., (**b**) 0.1% RL (**c**) 0.2% RL (**d**) 0.1% SL (**e**) 5% SL.

SEM analysis is key to understanding the morphological architecture of polymers and their behavior within heterogeneous compositions. The influence of different ratios of CARR and SA on the pore sizes of BLK composite wafers estimated from SEM analysis are shown in (Supplementary data Table S1). The morphology of 1.5% CARR:SA (1:3) BLK wafers (Figure 1a) showed a slanted interconnected network characterized by long leaves curled slightly at their tips with a combination of small round, keyhole shaped and large pores. The addition of 0.1% RL (Figure 1b) was observed to have transformed the slanted network of 1.5% CARR:SA (1:3) BLK wafers into larger and looser sheets dotted with a variety of small to medium sized pores with undefined shapes.

An increase in the amount of drug to 0.2% RL (Figure 1c), was however, observed to create a denser network interspersed with an even distribution of small oval and large sized pores. Addition of 0.1% SL (Figure 1d) transformed the slanted network of its corresponding BLK matrix into a vertical network of interconnected leafy-sheets embedded with small and large pores. The loading of 5% SL (Figure 1e) transformed the matrix into a uniform network of glassy leafs interspersed with equally large floral shaped pores. The loaded BSs were not visible on the wafer surface or pore walls, suggesting they were fully incorporated within the interior wafer matrix. The difference in the pore sizes of the selected optimized BLK and BSs loaded wafers was not statistically significant (*p* > 0.05).

### 3.3. Mechanical Strength (‘Hardness’)

Ideal mechanical properties of wound dressings include flexibility, durability, pliability, elasticity and resistance to stresses exerted by different parts of the body, particularly around the elbows and knees [39]. Wound dressings should withstand some frictional stresses during day-to-day activities when applied on the wound so that they can absorb the energy without breaking and thus provide a protective effect over the wound.

The ‘hardness’ is peak resistance force of the wafers to deformation and corresponds to the maximum force attained in the Texture Analyzer plot. With the exception of 1% CARR:SA (1:0) (Table 2) which showed similar hardness to 3% CARR:SA (1:0), the resistance to compression of pure CARR wafers was proportional to total polymer concentration. The events occurring involved an increase in resistance to compression from initial contact until the peak force when the maximum depth of compression was attained. Similarly, the resistance to compression of SA only wafers increased in accordance with total polymer weight 1% < 1.5% < 2%. Boateng and co-workers reported a peak force of 17.1(±2.1) N for 2% *w*/*w* SA [14], which is considerably higher than the value 5.02(±1.2) N obtained in this research. A possible explanation may be due to differences in mannuronic/guluronic ratios (M/G) of SA which in this investigation was 1.56 and affects mechanical properties as well as the sources of the alginates [40]. Additionally, their viscosities may be different as they were obtained from different manufacturers. Generally, guluronic acid side chains make gels and formulations harder while mannuronic acid chains make gels and subsequent formulations more flexible.

This difference was observed in the SEM micrographs where SA wafers [14] of the same magnification showed elongated pores unlike those reported in this research (Supplementary data Figure S1). Finally, the maximum depth of compression used in the study by Boateng and co was 2 mm compared to the 1 mm used in the current study.

The resistance to compression of 1–2% CARR:SA (1:1) composite wafers increased in accordance with total polymer weight, which was similar to the SA-only wafer. This similarity to the pure SA matrix may indicate that SA has a stronger influence on resistance to compression compared to CARR. However, the increased resistance to compression was less obvious in 1.5–2% CARR:SA (1:2) wafers. 1% CARR:SA (1:3) wafers had the lowest resistance to compression of all compositions containing a high SA content. An increase in mechanical strength beginning from 1% CARR:SA (1:3), 1.5% CARR:SA (1:3) formulations followed by doubled resistance from 1.5% CARR:SA (1:3), 2% CARR:SA (1:3) was observed. However, the hardness of 1.5% and 2% CARR:SA 2:1 and 3:1 composite wafers was comparable to that observed in CARR only wafers obtained from 1.5 and 2% gels, implying the dominance of CARR in these set of formulations.

Six composite formulations were observed to have potential ideal mechanical strength with resistance to compression ranging from 3.95 to 8.75 N. These formulations were 1.5% CARR:SA (1:1), 1.5% CARR:SA (1:2), 1.5% CARR:SA (1:3) and 2% CARR:SA (1:1), 2% CARR:SA (1:2) and 2% CARR:SA (1:3). However, comparison with their corresponding SEM micrographs (Supplementary data Figure S2) revealed similar surface morphologies which could be due to differences within the wafer matrix below the top surface. The microstructures of 1% CARR:SA (2:1), 1.5% CARR:SA (2:1) and 2% CARR:SA (2:1), 1% CARR:SA (3:1), 1.5% CARR:SA (3:1) and 2% CARR:SA (3:1) (Supplementary data Figure S3) were not taken forward for further characterization because they lacked the symmetry and interconnectivity of 1.5% CARR:SA (1:3) and 2% CARR:SA (1:2). The latter two formulations were therefore selected for bioanalytical evaluation of their fluid handling properties as part of the optimization process to select appropriate formulations for drug (BSs) loading. The mechanical strength of the optimized 1.5% CARR:SA (1:3) BLK wafers was higher than that of all the BSs-loaded wafers; however, the difference was statistically significant (*p* < 0.05) for only one of the BSs-loaded 1.5% CARR:SA (1:3) wafers.

### 3.4. X-ray Diffraction (XRD)

In this research, XRD was used to determine the crystalline or amorphous properties of representative formulated wafers. Five peaks were observed for pure CARR wafers (Supplementary data Figure S4). CARR showed an amorphous nature with the presence of additional peaks at 28.39 and 40.58, which may be attributed to inorganic potassium salt impurities such as KCl [41], whereas with the exception of one peak observed in pure SA wafers around 2-theta of 20°, no sharp peaks were observed which confirms the amorphous nature of SA (Supplementary data Figure S5).

**Figure 2:**
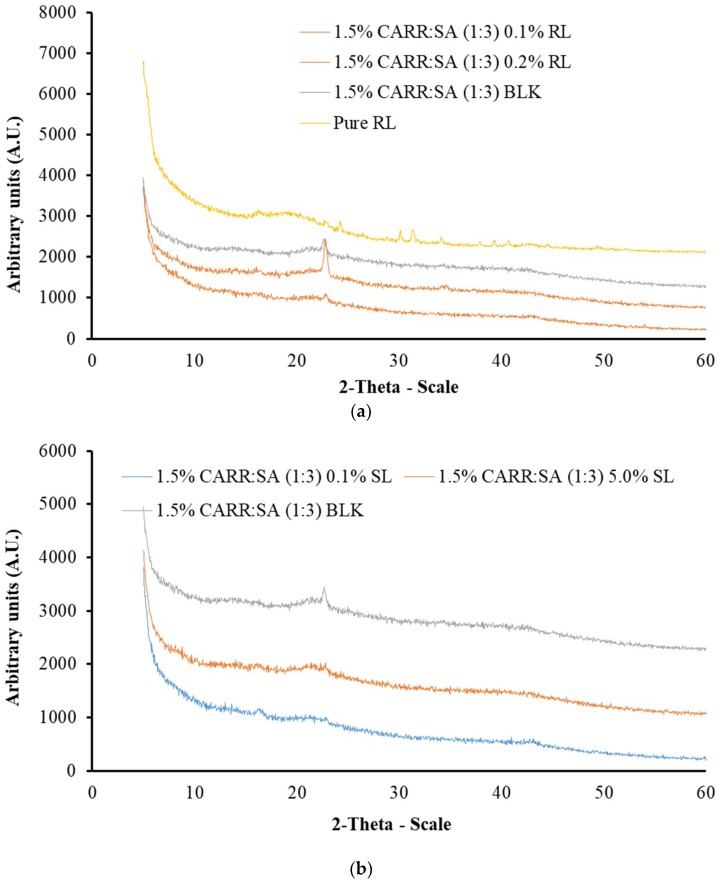
XRD diffractograms of (**a**) BLK and pure RL and RL loaded 1.5% CARR:SA (1:3) wafers and (**b**) BLK 1.5% CARR:SA (1:3) and SL loaded 1.5% CARR:SA (1:3) wafers.

These results agree with observations reported by Pawar et al. [19], in which two peaks were reported for pure CARR of the same grade. The diffractograms of 1% CARR:SA (1:2), 1% CARR:SA (1:2) and 1% CARR:SA (1:3) (Supplementary data Figure S6) were amorphous, with the exception of 1% CARR:SA (1:2) in which a crystalline ridge was observed. A very intense crystalline peak was observed in the matrix of 1.5% CARR:SA (1:3) which was between 20 and 30° 2-theta scale (Supplementary data Figure S7). The diffractograms of 2% CARR:SA (1:1), 2% CARR:SA (1:2) and 2% CARR:SA (1:3) mirrored the predominantly amorphous observations observed in corresponding 1% CARR:SA wafers (Supplementary data Figure S8). Although the wafers were also amorphous, the crystalline impurities were more observable in higher ratios of CARR:SA (2:1) as well as (3:1) (Supplementary data Figures S9–S11). The crystallinity of pure RL standard was in agreement with its corresponding diffractogram; however, pure SL was not analyzed by XRD due to its liquid amorphous state. However, the BSs-loaded wafers (Figure 2a,b) showed amorphous patterns which suggests that the RL was molecularly dispersed within the wafer matrix.

The physical form (crystalline or amorphous) of polymeric formulations affects various properties including water uptake, biodegradability and bioadhesion of the polymers [42]. The molecular dispersion of BSs in CARR:SA wafers will have high surface energy due to less ordered amorphous structures than the semi-crystalline form. Additionally, the decreased crystallinity of BSs could help to improve characteristics such as exudate absorption. Similar results were also reported by Pawar and co-workers [19] who reported that wafers prepared from two sets of polymeric blends (i) polyethylene oxide (POL) and carrageenan (CARR) (ii) POL and sodium alginate (SA) showed decreased crystallization of POL. They concluded that this may help to improve the properties stated above as well as the performance of the dressings such as prolonged retention at wound site, which can ultimately increase the bioavailability of the drug and reduce the need for a frequent change of dressing. Amorphous matrices allow greater molecular interaction between solutes and solvents, hence BSs are more soluble and dressings are expected to release BSs more quickly to stimulate pro-inflammatory cytokines and neutrophils and subsequently promote wound healing. However, this will need to be confirmed in an in vivo animal study.

### 3.5. Attenuated Total Reflectance Fourier Transform Infrared Spectroscopy (ATR-FTIR) 

FTIR spectral analysis of polymeric formulations such as freeze-dried wafers is a direct method used to evaluate molecular interactions such as hydrogen bonding or complexation by monitoring the band shift of a given functional group [43]. Hydrogen bonds are formed between the proton-donor and proton-acceptor molecules which shifts the bands to lower wavenumbers [44]. FTIR spectroscopy was employed to characterize the structure and the possible interactions in CARR and SA networks in comparison with their pure standards. Both CARR and SA are polyelectrolytes tend to form physical hydrogels with mono and polyvalent metallic cations [45].

**Figure 3:**
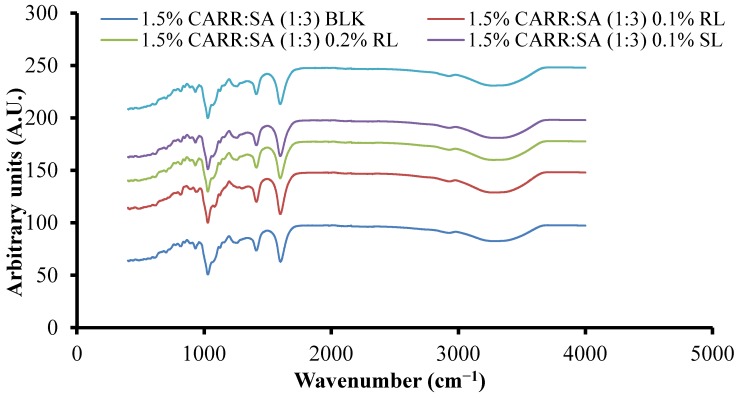
ATR-FTIR spectra of 1.5% CARR:SA (1:3) BLK and BSs loaded 1.5% CARR:SA (1:3) wafers.

Supplementary data (Table S2) show the FTIR spectra of CARR, SA, BSs and representative composite wafers. The peaks observed at 3373, 1221, 1037, 1157, 924 and 844 cm^−1^ can be attributed to O–H stretching, sulphate ester, C–O stretching of pyranose ring, 3,6-anhydro-d-galactose and, galactose 4-sulphate of CARR, respectively. All five peaks detected in pure CARR were present in 1–3% CARR:SA (1:0) wafers (data not shown). However, there was an additional peak at 1356–1374 cm^−1^ attributed to sulphate (SO_3_) group stretching in 1.5–3% CARR:SA (1:0) wafers. A peak attributed to C–O stretching of pyranose ring was observed in pure CARR powder and 1% CARR:SA (1:0). In the FTIR spectrum of SA powder, O–H stretching, asymmetric –COO– stretching, (C–OH deformation vibration) symmetric and (C–C stretching) were detected at vibrational frequencies of 3244, 1595, 1407 and 1025 cm^−1^, respectively. All four functional groups including C–O stretching at 1084 cm^−1^ were detected in 1–2% CARR:SA (0:1) wafers (data not shown). The influence of different ratios of CARR and SA on the shifting of FTIR characteristic bands is shown for representative formulations (Supplementary data Table S3). Although the combination of both polymers results in the formation of intermolecular bonds, the FTIR spectra of the different composite formulations were structurally closer to CARR or to SA depending on which one had a higher ratio within the wafer. Additionally, there appears to be very little difference in the wavenumbers of different BSs loaded in composite wafers as shown in Figure 3, indicating weak interactions between the BSs and composite polymers.

### 3.6. Fluid Handling Properties

The ability of wound dressings to absorb exudates and also provide a moist wound environment is necessary for the healing process. This biological function can be analyzed by water uptake as well as water loss characteristics as discussed below.

#### 3.6.1. Swelling

**Figure 4:**
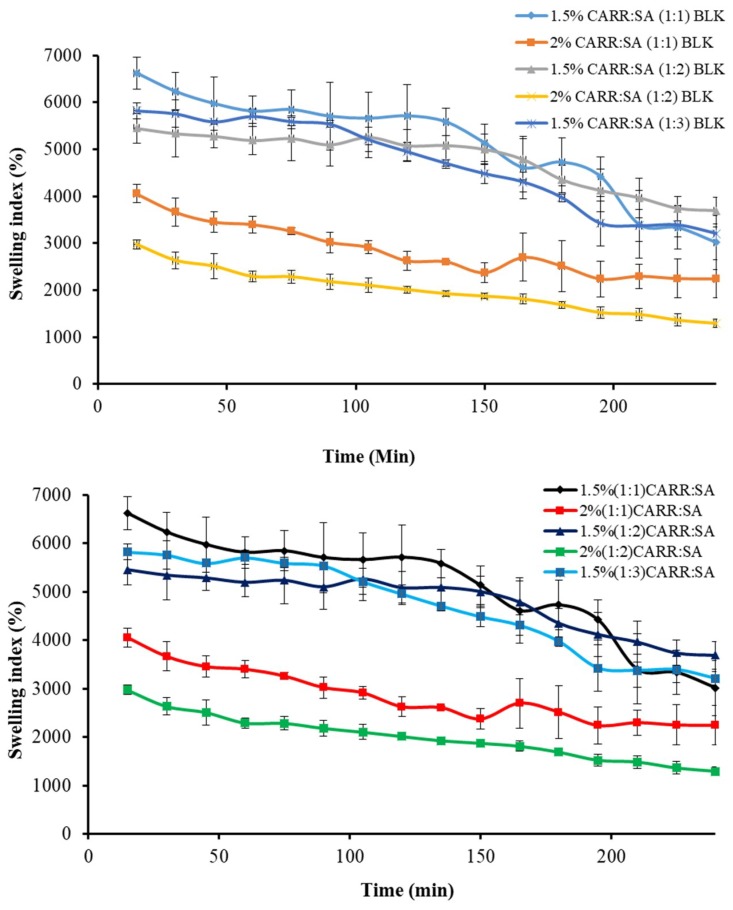
Swelling profiles in the presence of SWF, pH 7.5 for BLK CARR:SA (1:1 and 1:2 ratios) wafers prepared from 1.5 and 2% total polymer gels and CARR:SA 1:3 ratio wafers prepared from 1.5% gels.

The driving force for swelling is the entropy increase when water diffuses into the polymeric matrix. A formulation prepared from hydrophobic polymers has a non-favored interaction with water and does not swell much, while formulations prepared from hydrophilic polymers such as CARR and SA absorb large amounts of water and consequently swell to a greater extent. Swelling brings about tension within the gel network by stretching it and hence, lowers the entropy. Swelling also has an impact on drug release and a complex interaction between diffusion, dissolution and erosion mechanisms has been used to explain drug release from most hydrophilic matrices [46]. Fluid uptake (swelling) of the preliminary wafers prepared from 1% to 3% gels of only CARR showed erosion in less than one hour, which led to the decision to formulate composite wafers comprising CARR and SA polymers. During the preliminary swelling studies, the swelling capacity was observed to increase with increasing concentration of SA. However, the swelling of the samples reached the maximum value within 30 min of incubation in the swelling medium (Figure 4) after which the matrix begun to disintegrate. Among the different ratios of CARR:SA analyzed, maximum swelling capacity was observed in 1.5% CARR:SA (1:1). The 1.5% CARR:SA (1:3) BLK wafers showed maximum swelling capacity of 5825(±169)% which decreased to 2975(±94)% in 2% CARR:SA (1:2) BLK wafers, which may be due to the repulsive forces between the negatives charges of sulphate and carboxylate groups. Consequently, results obtained from swelling in conjunction with other characterization techniques led to the selection of 1.5% CARR:SA (1:3) for BSs loading.

**Figure 5:**
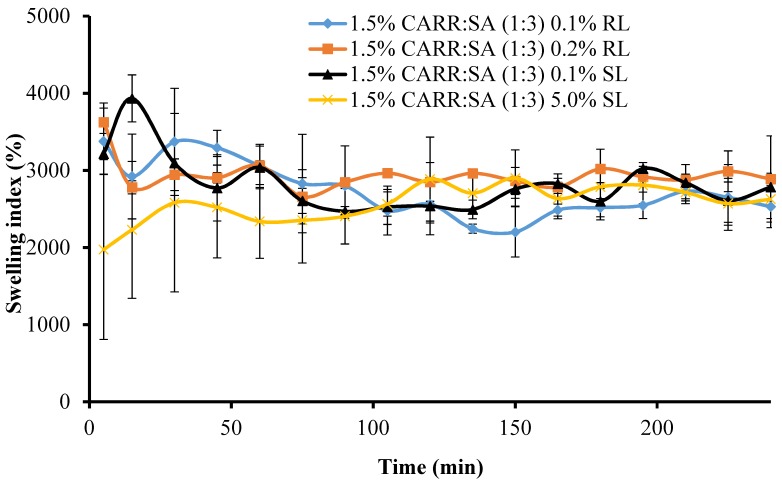
Swelling profiles in SWF, pH 7.5 of 1.5% CARR:SA (1:3) wafers loaded with 0.1% RL, 0.2% RL, 0.1% SL and 5% SL.

Figure 5 shows the change in swelling capacity (%) of the BSs loaded wafers with time. The maximum swelling capacities of 1.5% CARR:SA (1:3) loaded with 0.1% and 0.2% RL coincided with the wetting phase which occurred within the first 5 min.

There was a significant (*p* < 0.05) time gap between the maximum swelling capacities of wafers loaded with 0.1% and 5% SL (15 and 165 min, respectively). Additionally, 1.5% CARR:SA (1:3) 0.1% SL wafers had an overall maximum swelling capacity [3934(±305)%] for all the BSs-loaded wafers investigated in this study. It may be surmised from the results of the swelling investigation that 1.5% CARR:SA (1:3) wafers loaded with 0.1% BSs may be suitable for fast release. The difference in the wetting, maximum hydration and erosion (after 4 h) between BLK and BSs loaded wafers was statistically significant (*p* < 0.05) in only 1.5% CARR:SA (1:3) wafers. However, no statistical difference (*p* > 0.05) was observed between the different types and concentrations of BSs (0.1% RL 0.2% RL, 0.1% SL and 5% SL) loaded wafers, suggesting that incorporation of different amounts of the two BSs did not have significant effects on their physical properties as previously observed in the hardness and FTIR profiles.

#### 3.6.2. Pore Analysis

**Figure 6:**
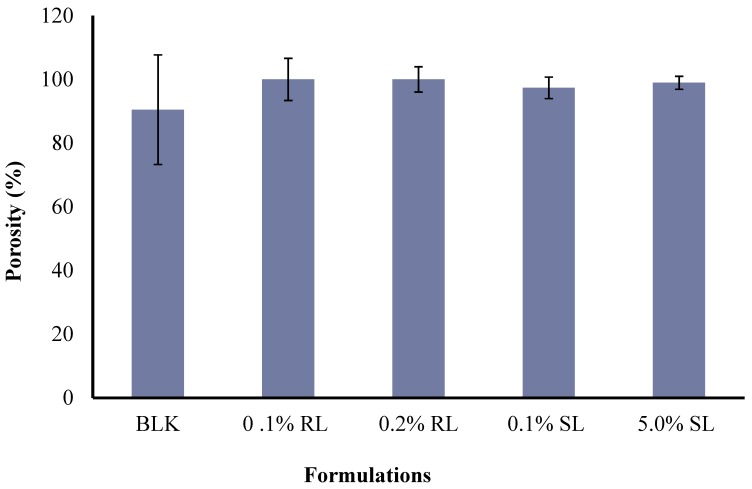
Comparison of the porosities of optimized BLK and BSs loaded composite wafers.

The porosities of BLK single polymer wafers, BLK composite CARR:SA wafers and BSs loaded wafers are reported in (Supplementary data Table S4). Overall, the maximum porosity of BLK formulations was observed in wafers prepared from 2% SA only gels with a value of 100%. This was followed by 1.5% CARR:SA (1:3) and 2% CARR:SA (1:2) wafers with porosities of 90.48% and 67.79%, respectively, whilst the lowest porosity occurred for 2% CARR:SA (1:1) wafers. The effect of a higher CARR ratio investigated at 2% CARR:SA (3:1) resulted in an observed percentage of 53.98%. It may therefore be stated that higher SA content enhances the porosity of composite wafers. BSs enhanced the porosity of the composite BLK wafer as all investigated formulations were above 97% (Figure 6).

Porous wound dressings have the advantage of higher exudate uptake. The difference in the pore analysis of BLK and BSs loaded wafers was statistically significant (*p* < 0.05) which does not agree with the SEM analysis, and this may be because the wafer pores created by lyophilization result in a complex architecture of void space which may have been displaced during the actual porosity measurements using the ethanol displacement method. However, no statistical difference (*p* > 0.05) was observed between the different BSs (0.1% RL, 0.2% RL, 0.1% SL and 5% SL)-loaded wafers.

#### 3.6.3. Water Absorption (*A*w) and Equilibrium Water Content (*EWC*)

The *A*w and *EWC* of wound dressings are important characteristics required for quick absorption of exudates.

**Figure 7:**
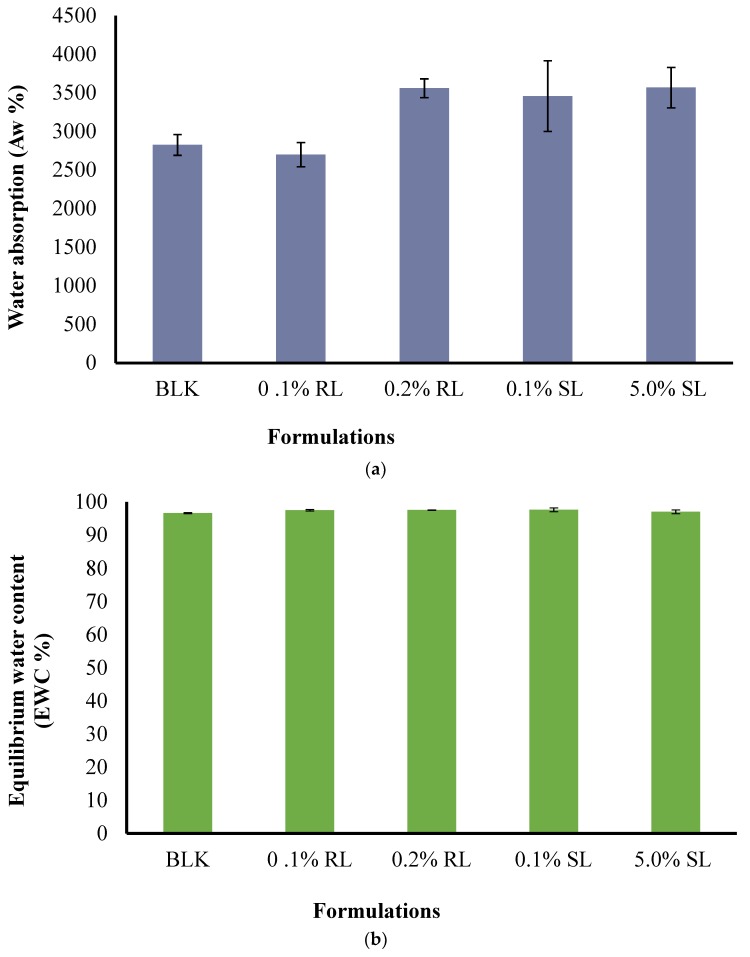
Comparison of (**a**) the water absorption (*A*w) and (**b**) the equilibrium water content (*EWC*) of optimized BLK and BSs loaded wafers.

The effect of CARR:SA ratios (Supplementary data Table S5) as well as BSs loading on *A*w and *EWC* were analyzed and reported for 1.5% CARR:SA (1:3) formulations in (Figure 7a,b). The *A*w of BLK 1.5% CARR:SA (1:3) composite wafer was observed as 3074(±241)% with *EWC* ≥ 97.00%, which indicates optimum stability of wafers after fluid uptake at body temperature (37 °C), while the *A*w of 1.5% CARR:SA (1:3) BSs-loaded wafers ranged from 2699(±157)–3569(±262)%. The difference in the (*A*w) and (*EWC*) of the selected optimized BLK and BSs loaded wafers was not statistically significant (*p* > 0.05), implying a negligible effect of BSs on the composite polymers.

#### 3.6.4. Evaporative Water Loss (*EWL*)

**Figure 8:**
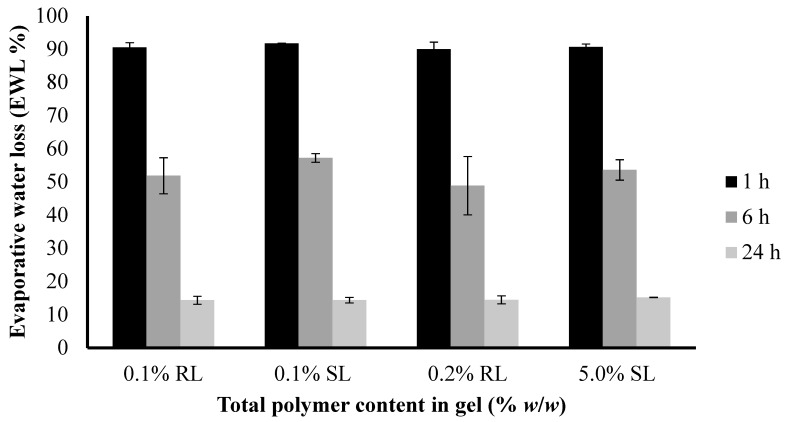
Comparison of the evaporative water loss (EWL) of optimized BLK and BSs loaded wafers.

The water loss from 1.5% CARR:SA (1:3) BLK and BSs-loaded wafers at 37 °C was compared to examine their potential behavior when used as a dressing over a chronic wound. As shown in (Figure 8), the loss of water ranged from about 9–10% after 1 h and increased up to 43–52% within 6 h (Supplementary data Table S6). After 24 h, the loss of water was about 85–86%.

The difference in the EWL of the selected optimized BLK and BSs loaded wafers was not statistically significant (*p* > 0.05). However, both dressings will lose water when exposed to air under dry conditions during short periods as a result of quick exudate uptake from the wound into the dressing and into the surrounding atmosphere by an active upward-directed process when used in early-stage exuding wounds [36]. These dressings may therefore be more beneficial to wounds which produce more exudates in early-stages of wound healing to avoid excessive collection of exudate which poses the risk of maceration of health surrounding skin.

#### 3.6.5. Water Vapor Transmission Rate

**Figure 9:**
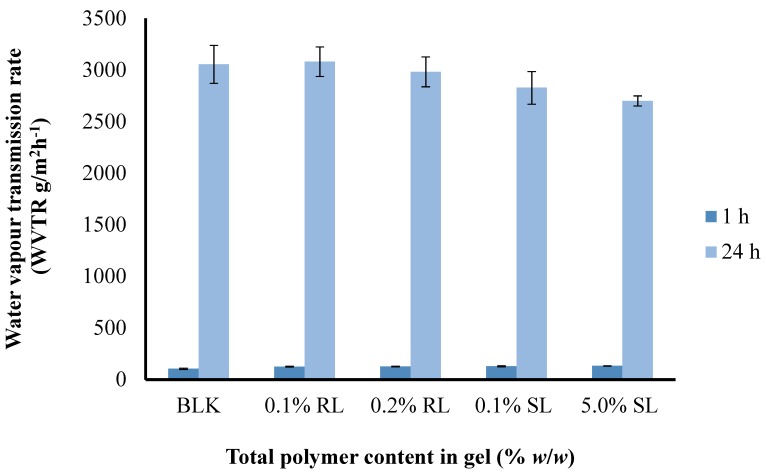
Comparison of the water vapor transmission rate (WVTR) of optimized BLK and BSs loaded wafers.

A moist environment enhances the healing process more effectively rather than a dry one [47]; therefore, a good dressing must be able to keep the wound environment moist to a certain level and must possess an appropriate WVTR. If the WVTR is fairly high, then there are chances of dehydration of wound as well as adherence of the dressing to wound bed. On the other hand, if the WVTR is fairly low then there may be excessive accumulation of exudate under the dressing which can cause maceration of healthy tissue or leakage of exudate from the edges of the dressing, which may promote microbial infection. The effect of CARR:SA ratios on the WVTR of BLK 1.5% (Figure 9) and 2% total polymer content wafers is presented in (Supplementary data Table S7).

The WVTR observed after 1 h ranged from (87.53–105.07) g/m^2^ h^−1^ and after 24 h from (2777–3082) g/m^2^ day^−1^ for 1.5% CARR:SA (1:3) BLK and BSs loaded wafers, respectively (Supplementary data Table S8). There was no statistical difference (*p* > 0.05) observed between the different BSs (0.1% RL, 0.2% RL, 0.1% SL and 5% SL)-loaded wafers prepared from the optimized 1.5% CARR:SA (1:3) gels. It has been recommended that a rate of 2000–2500 g/m^2^ day^−1^ would provide an adequate level of moisture without risk of wound dehydration [48]. Based on the above observations, it can be concluded that WVTR of all the samples falls above the prescribed range of WVTR; therefore, the BSs-loaded wafers in this study may be useful in the case of wounds with abnormally high exudates.

#### 3.6.6. Thermogravimetric Analysis (TGA)

TGA was used to estimate the amount of residual water present in single polymer and composite CARR:SA wafers prepared under similar conditions. The moisture content of single BLK and composite CARR:SA wafers at 1.5% and 2% total polymer content ranged from 13.75% and 15.16% (Supplementary data Table S9), which is an acceptable residual moisture for lyophilized wafers. Momoh and co-workers (2015) [49] reported a moisture content of 18.24% for pure SA which had the same mannuronic-guluronic (M/G) ratio of 1.56 as that used in this study M/G. The moisture content affects the exudate handling properties and improves the adhesion of the formulations.

#### 3.6.7. In Vitro Adhesion

**Figure 10:**
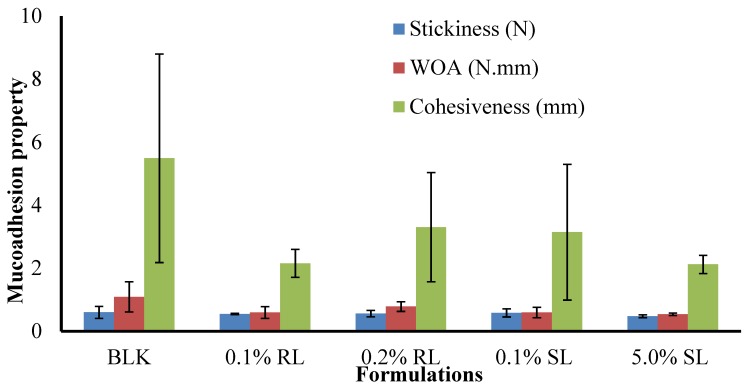
Comparison of the adhesive properties (stickiness, work of adhesion and cohesiveness) of BLK and DL composite 1.5% CARR:SA (1:3) wafers.

The stickiness, work of adhesion (WOA) and cohesiveness of BLK and BSs loaded wafers are presented in (Figure 10). The maximum stickiness observed for single CARR and SA wafers was 0.34 and 0.31 N, respectively, at 1% *w*/*w* total polymer content in gel. The stickiness of optimized 1.5% CARR:SA (1:3) was 0.60 N. Interestingly, the highest stickiness was observed in single 1.5% CARR wafers at 0.81 N. Generally, BSs reduced the stickiness of 1.5% CARR:SA (1:3).

The trends observed for the stickiness of BLK and BSs-loaded wafers were observed in the values recorded for WOA (Supplementary data Tables S10 and S11). Further, the trends observed for single CARR and SA as well as optimized composites were also observed in the cohesiveness of formulated wafers presented in (Supplementary data Table S12). Pawar et al. [19] associated the decreased stickiness of DL wafers in 2% BSA SWF to decreased porosity due to added drugs and subsequent sodium sulphate formation which inhibited the rapid hydration of the wafers. However, in this study, results obtained from pore analysis showed that the incorporation of drugs enhanced the porosities of the matrices which may result in increased hydration and drug release. Tobyn et al. [50] reported that increased ionic strength of the media and the presence of sodium and potassium ions results in decreased WOA.

The difference in the stickiness of BLK and BSs loaded wafers was not statistically significant (*p* > 0.05), while the difference in the WOA and cohesiveness of BLK and BSs loaded wafers was statistically significant (*p* < 0.05). Further, no statistical difference (*p* > 0.05) was observed between the different BSs (0.1% RL, 0.2% RL, 0.1% SL and 5% SL) loaded wafers. Cohesiveness is the intermolecular attraction which holds the wafer and the model wound substrate together. From the results obtained, it can be concluded that the wafers generally possessed good adhesive strength with the in vitro wound substrate. Furthermore, decreased stickiness and WOA is an advantage because it maintains a balance between prolonged retention at the wound site and the need to avoid damaging sensitive new tissue formed during the healing process in the course of dressing change.

## 4. Conclusions

The main objective of this investigation was the formulation design, development and optimization of stable composite polymer (CARR-SA)-based wafers loaded with BSs as potential dressings for application on chronic wounds. The results suggested that out of the five initial formulations, only composite wafers prepared from 1.5% CARR:SA (1:3) total polymer content in gel showed desirable characteristics required in wafers on the basis of an ideal balance between toughness (4–6N), porosity above 90% and optimum moisture handling properties which are essential functional characteristics. Molecular dispersity of all BSs was observed across all formulations with XRD diffractograms showing that all formulations were amorphous. The incorporation of BSs affected mechanical strength, exudate handling properties and mucoadhesion of optimized wafers. The seven functional groups identified in the ATR-FTIR spectra of BLK composite wafers were observed in corresponding BSs-loaded wafers, implying no interaction between the polymers and the loaded BSs. The wafers possessed ideal functional physico-chemical properties suitable for use as potential wound dressings for treating wounds and will be investigated further in future in vitro antibacterial and in vivo animal testing to confirm their efficacy.

## Figures and Tables

**Table 1:** Composition of polymers and BSs used in selected optimized freeze-dried wafers obtained from composite CARR:SA gels (1.5% *w*/*w* total polymer).

Starting Material	CARR:SA (BLK) (mg)	CARR:SA (0.1% RL) (mg)	CARR:SA (0.1% SL) (mg)	CARR:SA (0.2% RL) (mg)	CARR:SA (5% SL) (mg)
CARR	375.0	375.0	375.0	375.0	375.0
SA	1125.0	1125.0	1125.0	1125.0	1125.0
RL	-	1.5	1.5	-	-
SL	-	1.5	1.5	-	
RL	-	-	-	3.0	-
SL	-	-	-	-	75.0

**Table 2:** Hardness profiles of (**a**) BLK freeze-dried wafers prepared from different single polymer and composite gels at different total polymer concentrations (1–3%) and (**b**) BSs-loaded wafers prepared from 1.5% CARR:SA (1:3) composite gel (*n* = 4).

**(a)**							
**Gel Content (% *w*/*w*)**	**Hardness of BLK Single Wafers CARR:SA (N)**		**Hardness of BLK Composite Wafers CARR:SA (N)**				
1%	1:0	0:1	1:1	1:2	1:3	2:1	3:1
1	1.55(±0.2)	0.61(±0.1)	0.24(±0.1)	0.87(±0.1)	0.23(±0.0)	0.40(±0.0)	0.13(±0.0)
2	1.53(±0.2)	0.63(±0.2)	0.32(±0.1)	0.70(±0.2)	0.18(±0.0)	0.39(±0.0)	0.11(±0.0)
3	1.48(±0.2)	0.57(±0.1)	0.28(±0.0)	0.71(±0.2)	0.29(±0.0)	0.47(±0.1)	0.11(±0.0)
4	1.29(±0.2)	0.61(±0.1)	0.48(±0.1)	0.87(±0.2)	0.28(±0.0)	0.43(±0.1)	0.13(±0.0)
1.5%	1:0	0:1					
1	0.40(±0.1)	2.16(±0.2)	4.02(±0.6)	4.33(±0.4)	4.50(±0.6)	0.80(±0.1)	0.44(±0.1)
2	0.35(±0.1)	2.08(±0.3)	4.33(±0.5)	4.11(±0.4)	3.89(±0.6)	0.73(±0.1)	0.39(±0.1)
3	0.46(±0.1)	1.94(±0.3)	4.68(±0.1)	3.78(±0.5)	3.90(±0.6)	0.74(±0.1)	0.44(±0.1)
4	0.33(±0.1)	1.83(±0.4)	4.12(±0.6)	3.59(±0.4)	4.03(±0.8)	0.65(±0.1)	0.44(±0.1)
2%	1:0	0:1					
1	0.60(±0.1)	4.40(±1.0)	8.34(±1.5)	4.77(±0.3)	7.72(±1.3)	0.74(±0.2)	3.75(±0.2)
2	0.67(±0.2)	6.22(±1.1)	8.52(±0.5)	4.26(±0.5)	7.65(±0.8)	0.47(±0.1)	3.02(±0.3)
3	0.42(±0.1)	5.04(±1.0)	8.30(±0.5)	4.82(±0.5)	8.29(±1.3)	0.62(±0.3)	3.66(±0.4)
4	0.60(±0.1)	4.44(±1.0)	9.81(±0.4)	4.79(±0.5)	8.39(±1.0)	0.52(±0.1)	2.99(±0.3)
2.5%	1:0	0:1					
1	0.83(±0.2)	-	-	-	-	-	-
2	0.86(±0.0)	-	-	-	-	-	-
3	0.97(±0.1)	-	-	-	-	-	-
4	0.90(±0.1)	-	-	-	-	-	-
3%	1:0	0:1					
1	1.76(±0.3)	-	-	-	-	-	-
2	1.12(±0.2)	-	-	-	-	-	-
3	1.51(±0.2)	-	-	-	-	-	-
4	1.39(±0.3)	-	-	-	-	-	-
**(b)**							
	**1.5% (1:3) DL Wafers**		**0.1% RL**	**0.2% RL**	**0.1% SL**	**5% SL N**	
	1		2.77(±0.5)	2.78(±0.3)	2.86(±0.3)	3.43(±0.3)	
	2		2.63(±0.4)	2.81(±0.3)	2.94(±0.4)	3.14(±0.3)	
	3		2.60(±0.5)	3.09(±0.2)	3.18(±0.5)	3.40(±0.2)	
	4		2.81(±0.4)	3.09(±0.4)	2.97(±0.4)	3.93(±0.6)	

## Supplementary Materials

The following are available online at http://www.mdpi.com/2073-4360/10/8/918/s1, Table S1: Comparison of the mean pore sizes (± SD) (μm) of single, composite and optimized BSs-loaded wafers (n = 3), Table S2: Wavenumbers of various polymer and BSs starting materials and representative single CARR and SA wafers based on possible intermolecular/intramolecular interactions analyzed by ATR-FTIR analysis, Table S3: Comparison of wavenumbers present in selected optimized CARR:SA:BLKs and representative CARR:SA:BSs-loaded wafers based on ATR-FTIR analysis, Table S4: Comparison of the porosities of single, composite and BSs-loaded wafers, Table S5: Comparison of the water absorption (Aw) and equilibrium water content (*EWC*), of single, composite and BSs-loaded wafers, Table S6: Comparison of the Evaporative water loss (EWL) of 1.5% CARR:SA (1:3) BSs-loaded wafers, Table S7: Comparison of the water vapor transmission rate (WVTR) of BLK 1.5% and 2% CARR:SA (0:1, 1:1, 1:2, 1:3, 3:1) wafers, Table S8: Comparison of the water vapor transmission rate (WVTR) of BSs-loaded 1.5% CARR:SA (1:3) wafers, Table S9: Residual moisture of optimized formulations analyzed by TGA, Table S10: Comparison of the mucoadhesive stickiness of single, composite BLK and drug-loaded wafers, Table S11: Comparison of the work of adhesion (WOA) of single, composite and drug-loaded wafers, Table S12: Comparison of the mucoadhesive cohesiveness of single, composite and drug-loaded wafers, Figure S1: SEM comparison of selected single polymer wafers prepared from pure CARR (ai) 1%(1:0) (aii) 1.5%(1:0) (aiii) 2%(1:0) pure SA (bi) 1%(0:1) (bii) 1.5%(0:1) (biii) 2%(0:1) and higher total polymer weight pure CARR gels (ci) 2.5%(1:0) (cii) 3%(1:0), Figure S2: SEM images of composite wafers obtained from 1.0, 1.5 and 2.0 % (total polymer weight) CARR:SA gels at ratios of 1:0, 0:1, 1:1, 1:2 and 1:3 respectively, Figure S3: Comparison of (ai) 1%(2:1) (aii) 1.5%(2:1) (aiii) 2%(2:1) (bi) 1%(3:1) (bii)1.5%(3:1) (biii) 2%(3:1) CARR:SA wafers, Figure S4: XRD diffractograms of 1–3% CARR:SA (1:0) BLK, Figure S5: XRD diffractograms of 1–2% CARR:SA (0:1), Figure S6: XRD diffractograms of 1% CARR:SA (1:1, 1:2 and 1:3), Figure S7: XRD diffractograms of 1.5%(1:1, 1:2 and 1:3) CARR:SA, Figure S8: XRD diffractograms of 2% CARR:SA (1:1, 1:2 and 1:3) BLK, Figure S9: XRD diffractograms of 1% CARR:SA (2:1) BLK and 1.5% CARR:SA (2:1) BLK, Figure S10: XRD diffractograms of 1% CARR:SA (3:1) BLK and 1.5% CARR:SA (3:1) BLK, Figure S11: XRD diffractogram of 2% CARR:SA (2:1).
