# Supplementary material for: Composite Biopolymer-Based Wafer Dressings Loaded with Microbial Biosurfactants for Potential Application in Chronic Wounds
Source: Polymers (Basel). 2018 Aug 15;10(8):918. doi: 10.3390/polym10080918 (PMC6403717; doi:10.3390/polym10080918)
Supplement: Supplementary file 1 [file polymers-10-00918-s001.pdf]

Composite Biopolymer-based Wafer Dressings Loaded with Microbial Biosurfactants for Potential Application in Chronic Wounds

Olufunke Akiyode[1] and Joshua Boateng[1*]

[1] Department of Pharmaceutical, Chemical and Environmental Sciences, Faculty of Engineering and Science, University of Greenwich, Medway, Kent, UK, ME4 4TB

*Correspondence: Dr Joshua Boateng (j.s.boateng@gre.ac.uk; joshboat40@gmail.com).

SUPPLEMENTARY TABLES

Table S1. Comparison of the mean pore sizes (± SD) (μm) of single, composite and optimized BSs loaded wafers (*n* = *3*).

(% w/w)	1:0	0:1	(μm)	
1.0	173.77 (± 75.99)	126.10 (± 45.04)	-	-
1.5	146.56 (± 53.65)	85.63 (± 25.64)	-	-
2.0	79.06 (± 23.22)	65.78 (± 13.90)	-	-
2.5	90.66 (± 28.08)	-	-	-
3.0	97.88 (± 22.35)	-	-	-
	1:1	1:2	1:3	3:1
1.5	87.07 (± 30.26)	169.00 (± 38.66)	78.80 (± 15.92)	-
2.0	180.25 (± 53.44)	87.10 (± 19.37)	89.07 (± 22.22)	153.80 (± 34.30)
	0.1% RL	0.2% RL	0.1% SL	5% SL
1.5	136.60 (± 50.95)	122.26 (± 46.30)	98.21 (± 42.52)	141.13 (± 47.15)

Table S2. Wavenumbers of various polymer and BSs starting materials and representative single CARR and SA wafers based on possible intermolecular/intramolecular interactions analyzed by ATR-FTIR analysis.

Peak number	CARR STD	SA	RL	SL	1.5% (1:0)	1.5% (0:1)
1	3373	3244	3257	3368	3368	3252
2	-	1595	2924-2855	2928-2855	1373	1598
3	1221	1407	1726	-	1224	1408
4	-	-	1655	1641	1156	1083
5	925	1025	1575	1553	925	1028
6	844	-	-	1415	844	-
7	-	-	1397	1369	-	-
8	-	-	1317	1247	-	-
9	-	-	1123	1170	-	-
10	-	-	-	1077	-	-
11	-	-	1046	1034	-	-
12	-	-	981		-	-
13	-	-	916	-	-	-
14	-	-	881	-	-	-
15	-	-	831	-	-	-
16	-	-	808	-	-	-
17	-	-	704	-	-	-
18	-	-	663	-	-	-

Table S3. Comparison of wavenumbers present in selected optimised CARR:SA:BLKs and representative CARR:SA:BSs loaded wafers based on ATR-FTIR analysis

Peak number	1.5% (1:3)	1.5% (1:3) 0.2% RL	1.5% (1:3) 0.1% SL
1	3337	3283	3284
2	1599	1598	1599
3	1410	1411	1411
4	1255	1255	1255
5	1029	1028	1029
6	931	931	932
7	-	846	-

Table S4. Comparison of the porosities of single, composite and BSs loaded wafers

(%, w/w)	CARR :SA 0:1	Pore analysis (%)		
1.5	-			
2.0	100.00 (±7.20)			
	1:1	1:2	1:3	3:1
1.5	49.92 (± 11.10)	50.83 (± 1.40)	90.48 (± 17.20)	-
2.0	43.26 (± 10.21)	67.79 (± 15.10)	49.25 (± 5.04)	53.98 (± 7.25)
	0 .1% RL	0.2% RL	0.1% SL	5% SL
1.5	100.00 (± 6.62)	100.00 (± 3.97)	97.34 (± 3.37)	98.94 (± 2.04)

Table S5. Comparison of the water absorption (*A*w) and equilibrium water content (EWC), of single, composite and BSs loaded wafers

1.5	0:1	1:1	1:2	1:3	3:1
*A*w	-	2521 (± 468)	3074 (± 241)	2826 (± 135)	-
EWC	-	96.12 (± 0.63)	96.84 (± 0.23)	96.58 (± 0.16)	-
2	0:1	1:1	1:2	1:3	3:1
*A*w	2369 (± 75)	1974 (± 180)	2574 (± 590)	1943 (± 412)	2854 (± 407)
EWC	95.95 (± 0.12)	95.15 (± 0.43)	96.14 (± 0.84)	94.95 (± 1.15)	96.57 (± 0.44)
1.5	0 .1% RL	0.2% RL	0.1% SL	5% SL	
*A*w	2699 (± 157)	3560 (± 122)	3458 (± 458)	3569 (± 262)	-
EWC	97.46 (± 0.24)	97.53 (± 0.05)	97.61 (± 0.57)	97.00 (± 0.57)	-

Table S6. Comparison of the evaporative water loss (EWL) of 1.5% CARR:SA (1:3) BSs loaded wafers

1.5%(1:3) CARR:SA	1	2	3	4	5	6	24 h (%)
0.1% RL	90.54 (± 1.41)	81.05 (± 2.48)	72.11 (± 3.69)	64.56 (± 4.38)	57.83 (± 4.99)	51.86 (± 5.43)	14.36 (± 1.20)
0.1% SL	91.70 (± 0.13)	83.44 (± 0.32)	75.32 (± 0.50)	68.53 (± 0.76)	62.49 (± 1.24)	57.24 (± 1.29)	14.40 (± 0.84)
0.2% RL	89.99 (± 2.13)	79.87 (± 4.08)	70.17 (± 5.77)	62.15 (± 6.91)	55.10 (± 7.89)	48.87 (± 8.78)	14.48 (± 1.21)
5% SL	90.63 (± 0.90)	81.50 (± 1.59)	72.90 (± 2.13)	65.66 (± 2.47)	59.44 (± 2.74)	53.62 (± 3.07)	15.22 (± 0.03)

Table S7. Comparison of the water vapour transmission rate (WVTR) of BLK 1.5 and 2% CARR:SA (0:1, 1:1, 1:2, 1:3, 3:1) wafers

1.5%	1	2	3	4	24 h (g/m^2day^{-1})
0:1	-	-	-	-	-
1:1	87.53 (± 4.28)	199.76 (± 4.27)	317.66 (± 6.61)	595.70 (± 50.41)	3082 (± 285)
1:2	89.03 (± 1.31)	201.27 (± 3.46)	318.79 (± 4.61)	644.94 (± 24.17)	2777 (± 105)
1:3	105.07 (± 6.21)	250.13 (± 57.90)	376.89 (± 69.67)	735.29 (± 76.06)	3054 (± 184)
3:1	-	-	-	-	-
2%					
0:1	124.12 (± 19.57)	225.42 (± 24.83)	346.33 (± 30.39)	660.59 (± 49.66)	2920 (± 132)
1:1	84.32 (± 3.71)	189.58 (± 7.09)	300.68 (± 9.63)	585.14 (± 13.90)	2661 (± 114)
1:2	89.41 (± 2.59)	198.63 (± 2.04)	311.24 (± 2.04)	577.78 (± 4.63)	2657 (± 69)
1:3	130.72 (± 63.72)	318.04 (± 103.78)	491.77 (± 167.47)	886.95 (± 219.86)	3285 (± 690)
3:1	130.72 (± 41.68)	260.88 (± 44.32)	386.70 (± 45.70)	731.14 (± 112.37)	3330 (± 439)

Table S8. Comparison of the water vapour transmission rate (WVTR) of BSs loaded 1.5% CARR:SA (1:3) wafers

1.5% CARR:SA (1:3)	1	2	3	4	5	6	24 h (g/m^2day^{-1})
0.1% RL	126 (±5)	278 (±5)	615 (±20)	832 (±45)	990 (±56)	1148 (±66)	3080 (±143)
0.2% RL	127 (±3)	277 (±9)	590 (±27)	806 (±37)	958 (±51)	1106 (±61)	2982 (±145)
0.1% SL	129 (±5)	275 (±9)	498 (±36)	672 (±53)	818 (±56)	965 (±67)	2828 (±157)
5% SL	133 (±2)	273 (±5)	433 (±38)	586 (±38)	725 (±37)	864 (±38)	2702 (±49)

Table S9. Residual moisture of optimized formulations analyzed by TGA

Moisture content (%)	0:1	1:1	1:2	1:3	3:1
1.5%	-	14.18 (±0.64)	14.46 (±0.10)	14.86 (±0.53)	-
2.0%	14.36 (±0.06)	13.75 (±0.33)	14.23 (±0.10)	15.16 (±0.18)	13.77 (±1.33)

Table S10. Comparison of the mucoadhesive stickiness of single, composite BLK and drug loaded wafers

Stickiness (N)					
Single polymer wafers	1:0	0:1			
1 (% w/w)	0.34 (± 0.12)	0.31 (± 0.02)	-	-	-
1.5 (% w/w)	0.14 (± 0.02)	0.28 (± 0.06)	-	-	-
2 (% w/w)	0.16 (± 0.01)	0.29 (± 0.02)	-	-	-
2.5 (% w/w)	0.18 (± 0.03)	-	-	-	-
3 (% w/w)	0.20 (± 0.00)	-	-	-	-
Composite wafers	1:1	1:2	1:3	2:1	3:1
1 (% w/w)	0.29 (± 0.11)	0.36 (± 0.14)	0.24 (± 0.02)	0.26 (± 0.09)	0.18 (± 0.04)
1.5 (%, w/w)	0.81 (± 0.06)	0.47 (± 0.14)	0.60 (± 0.19)	-	-
2 (% w/w)	0.42 (± 0.09)	0.35 (± 0.11)	0.61 (± 0.22)	-	0.47 (± 0.17)
DL loaded wafers	0.1% RL	0.2% RL	0.1% SL	5% SL	-
1:3 (1.5%)	0.55 (± 0.02)	0.56 (± 0.10)	0.58 (± 0.13)	0.48 (± 0.05)	-

Table S11. Comparison of the work of adhesion (WOA) of single, composite and drug loaded wafers

Work of adhesion (WOA) (N.mm)					
Single polymer wafers	1:0	0:1			
1.0 (% w/w)	0.61 (± 0.20)	0.37 (± 0.03)	-	-	-
1.5 (% w/w)	0.23 (± 0.00)	0.35 (± 0.10)	-	-	-
2.0 (% w/w)	0.30 (± 0.02)	0.22 (± 0.04)	-	-	-
2.5 (% w/w)	0.41 (± 0.19)	-	-	-	-
3 .0(% w/w)	0.23 (± 0.01)	-	-	-	-
Composite wafers	1:1	1:2	1:3	2:1	3:1
1.0 (% w/w)	0.38 (± 0.17)	0.72 (± 0.37)	0.45 (± 0.02)	0.23 (± 0.11)	0.30 (± 0.03)
1.5 (% w/w)	1.19 (± 0.12)	0.68 (± 0.31)	1.09 (± 0.48)	-	-
2.0 (% w/w)	0.47 (± 0.20)	0.54 (± 0.21)	1.91 (± 1.99)	-	0.67 (± 0.24)
DL loaded wafers	0.1% RL	0.2% RL	0.1% SL	5% SL	-
1:3 (1.5%)	0.59 (± 0.19)	0.78 (± 0.15)	0.59 (± 0.17)	0.54 (± 0.04)	

Table S12. Comparison of the mucoadhesive cohesiveness of single, composite and drug loaded wafers

Cohesiveness (mm)					
Single wafers	1:0	0:1	-	-	-
1 (% w/w)	3.61 (± 0.51)	2.92 (± 1.21)	-	-	-
1.5 (% w/w)	3.01 (± 0.33)	2.40 (± 0.54)	-	-	-
2 (% w/w)	4.03 (± 1.36)	1.61 (± 0.49)	-	-	-
2.5 (% w/w)	4.90 (± 2.38)	-	-	-	-
3 (% w/w)	2.29 (± 0.09)	-	-	-	-
Composite wafers	1:1	1:2	1:3	2:1	3:1
1 (% w/w)	2.05 (± 0.28)	3.14 (± 0.67)	3.62 (± 0.45)	1.50 (± 0.39)	3.50 (± 0.57)
1.5 (% w/w)	3.61 (± 0.43)	2.84 (± 0.86)	5.49 (± 3.31)	-	-
2 (% w/w)	2.16 (± 0.38)	2.98 (± 0.80)	4.91 (± 2.87)	-	3.30 (± 0.88)
DL loaded wafers	0.1% RL	0.2% RL	0.1% SL	5% SL	-
1.5%(1:3)	2.15 (± 0.44)	3.30 (± 1.73)	3.14 (± 2.15)	2.12 (± 0.29)	-

SUPPLEMENTARY FIGURES

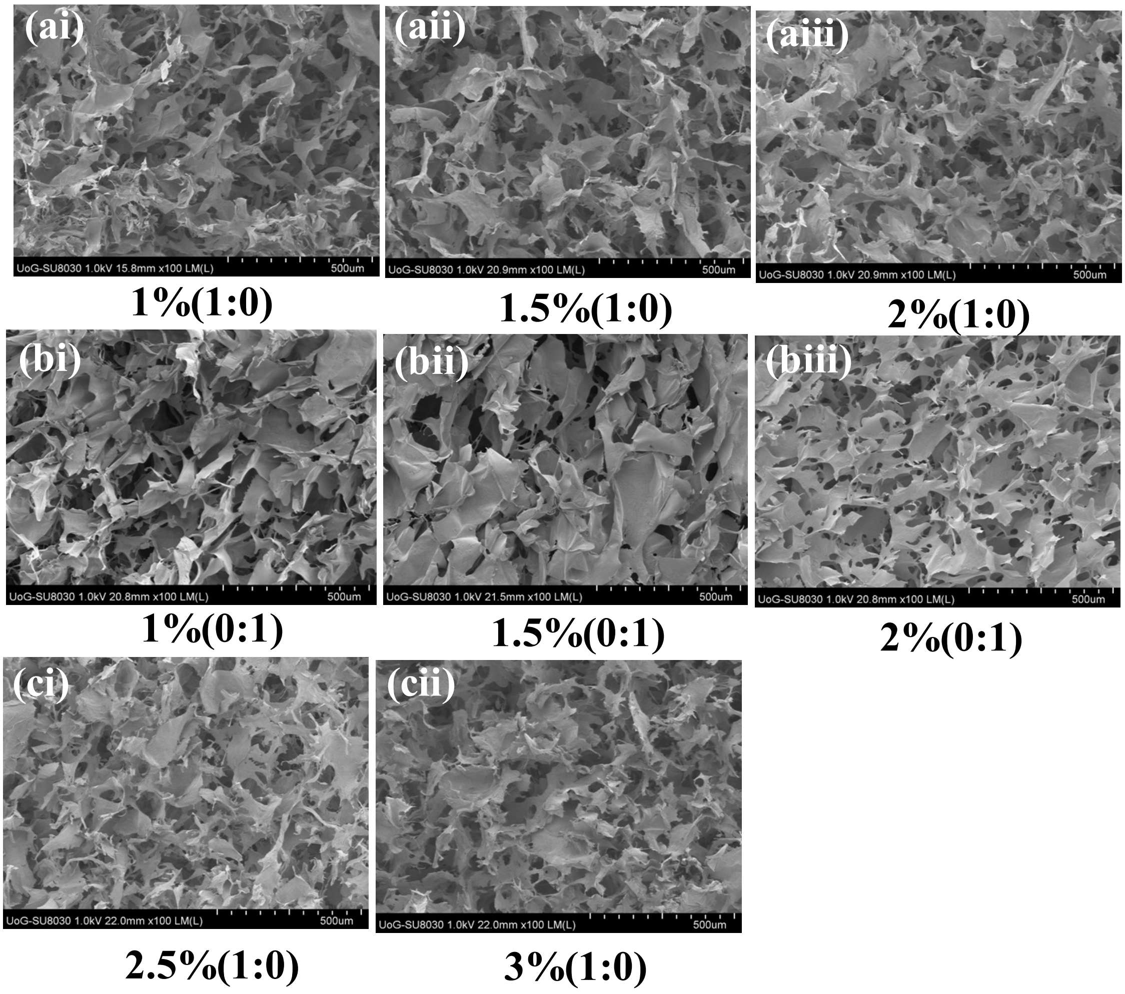

Figure S1. SEM comparison of selected single polymer wafers prepared from pure CARR (ai) 1%(1:0) (aii) 1.5%(1:0) (aiii) 2%(1:0) pure SA (bi) 1%(0:1) (bii) 1.5%(0:1) (biii) 2%(0:1) and higher total polymer weight pure CARR gels (ci) 2.5%(1:0) (cii) 3%(1:0).

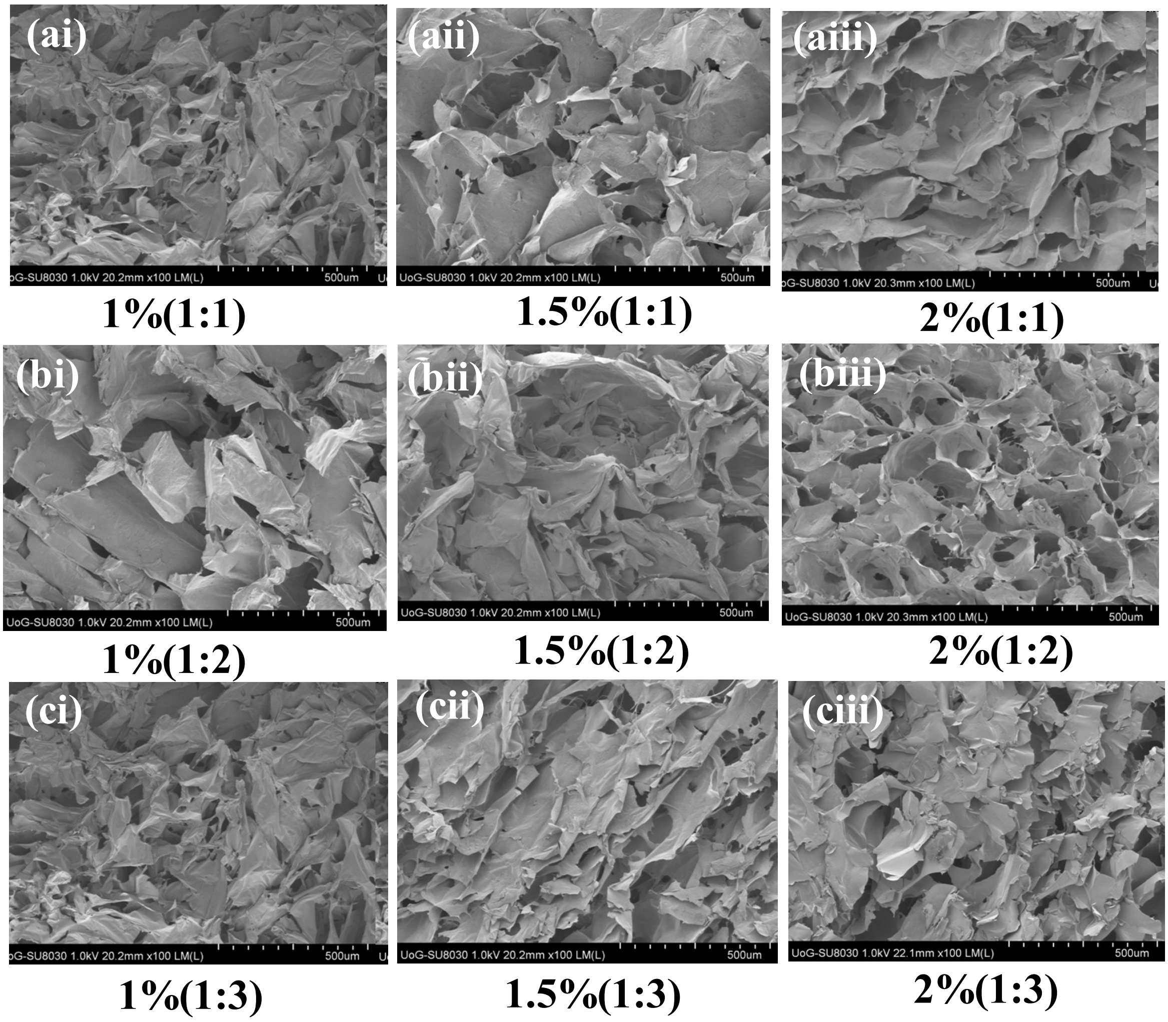

Figure S2. SEM images of composite wafers obtained from 1.0, 1.5 and 2.0 % (total polymer weight) CARR:SA gels at ratios of 1:0, 0:1, 1:1, 1:2 and 1:3 respectively.

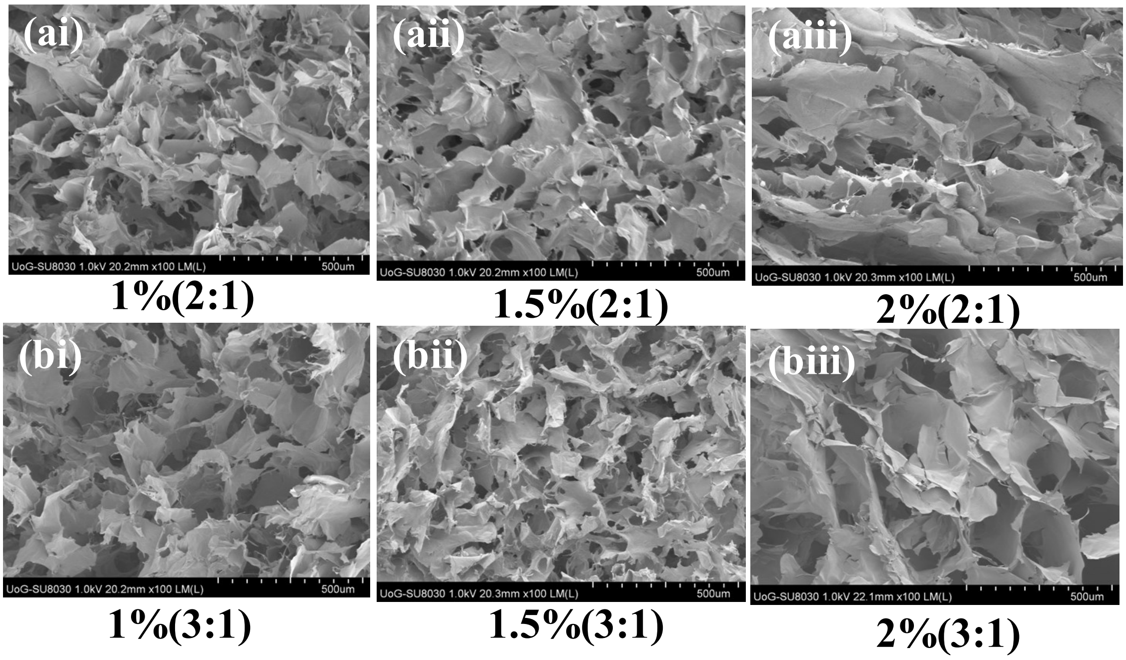

Figure S3. Comparison of (ai) 1%(2:1) (aii) 1.5%((2:1) (aiii) 2%(2:1) (bi) 1%(3:1) (bii)1.5%(3:1) (biii) 2%(3:1) CARR:SA wafers.

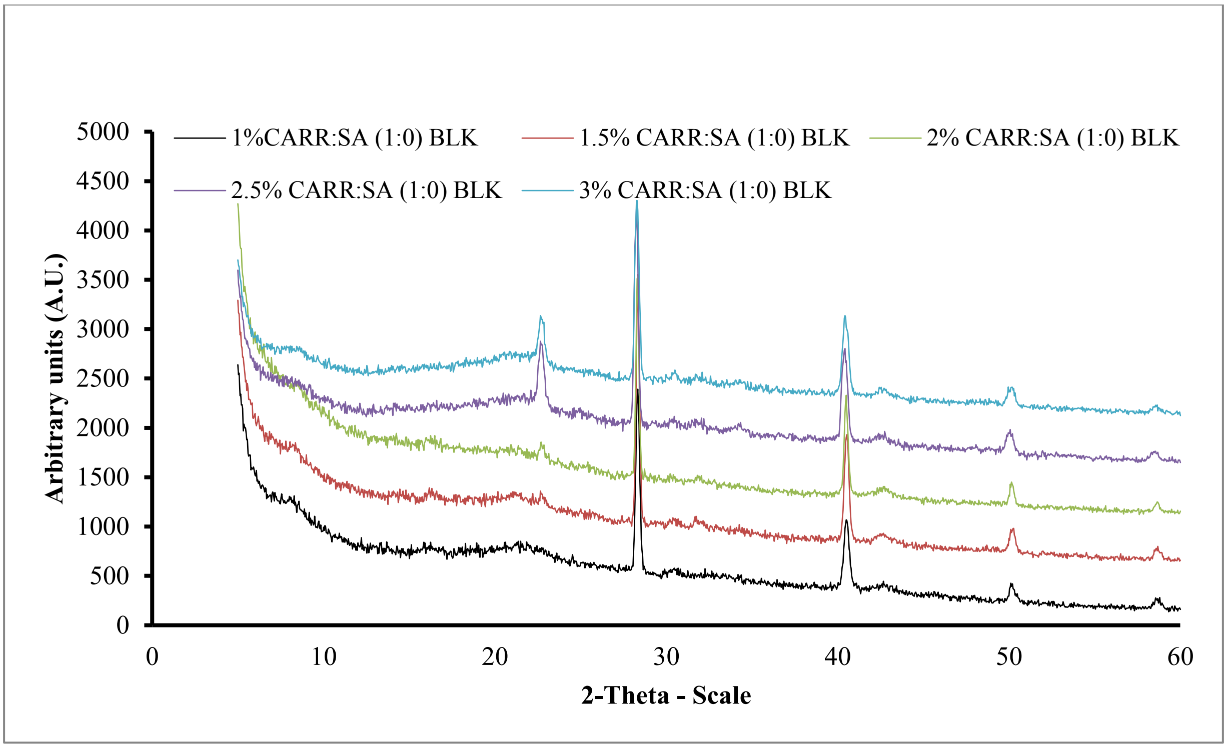

Figure S4. XRD diffractograms of 1 - 3% CARR:SA (1:0) BLK.

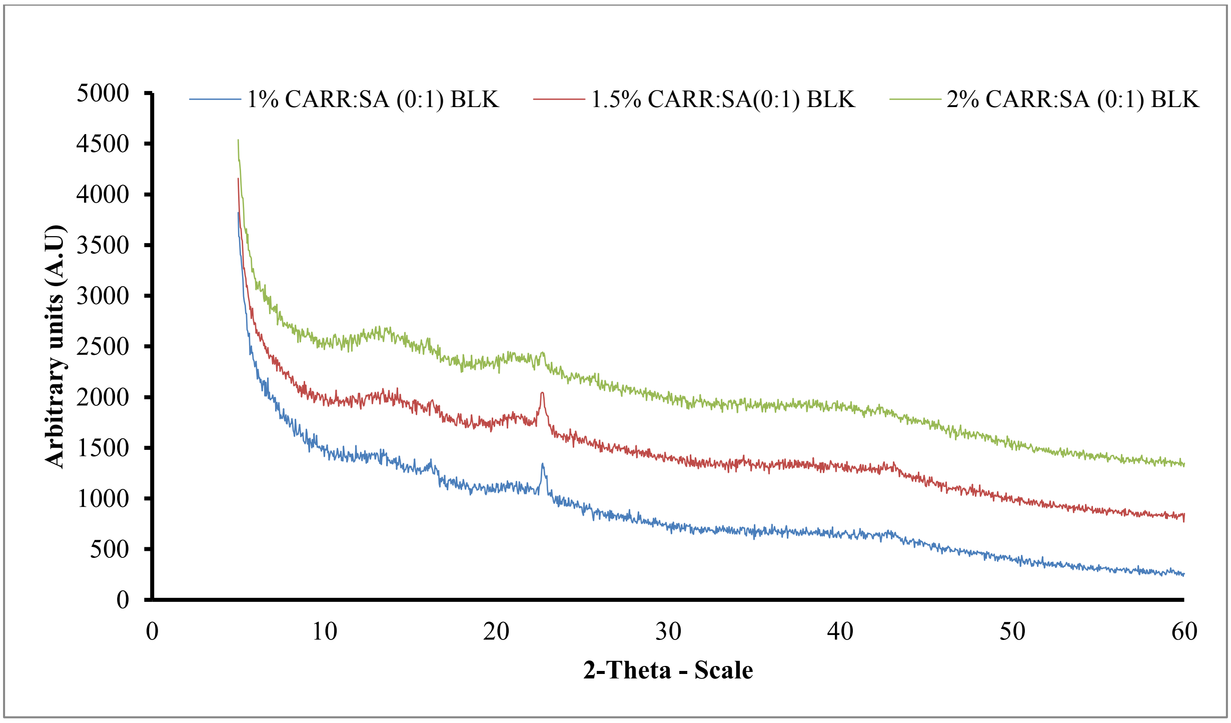

Figure S5. XRD diffractograms of 1 - 2% CARR:SA (0:1).

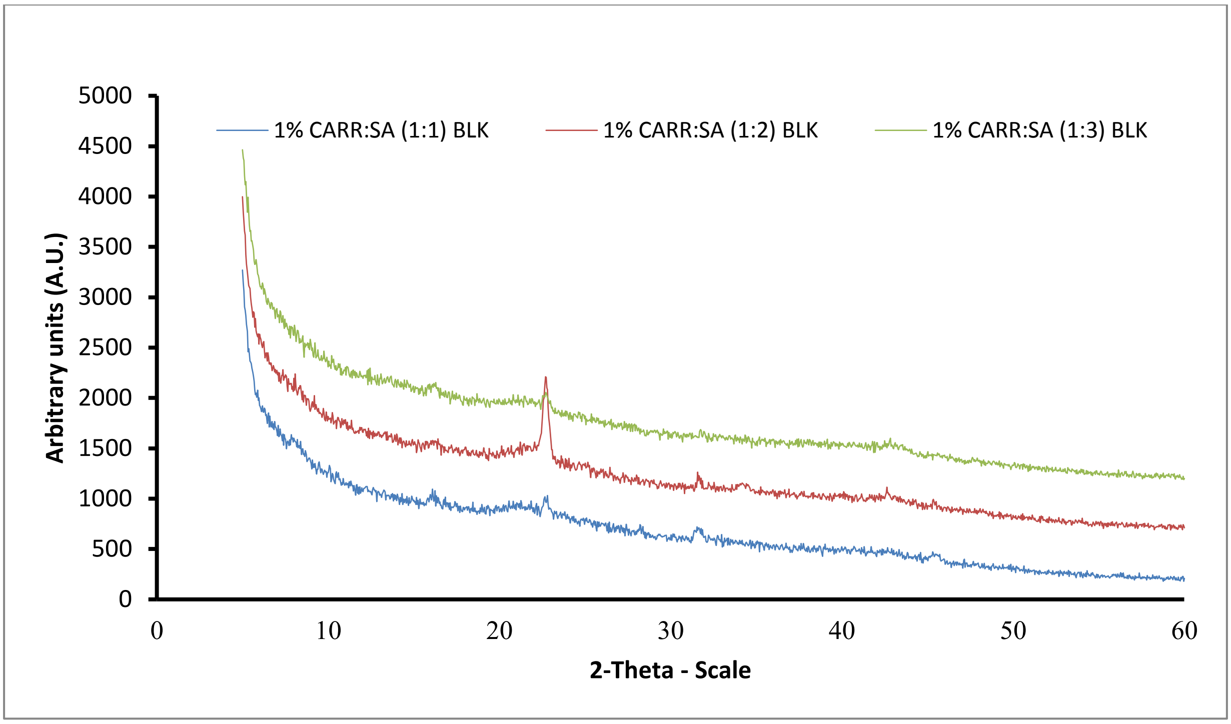

Figure S6. XRD diffractograms of 1% CARR:SA (1:1, 1:2 and 1:3).

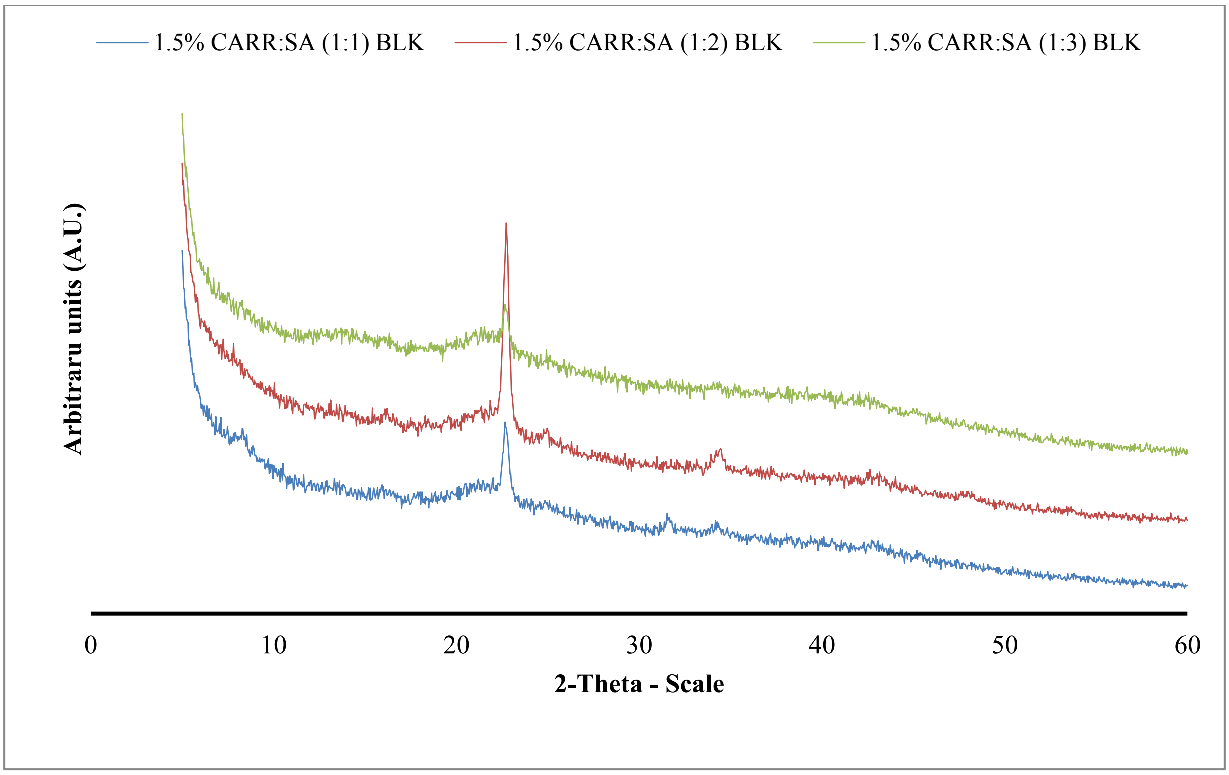

Figure S7. XRD diffractograms of 1.5%(1:1, 1:2 and 1:3) CARR:SA.

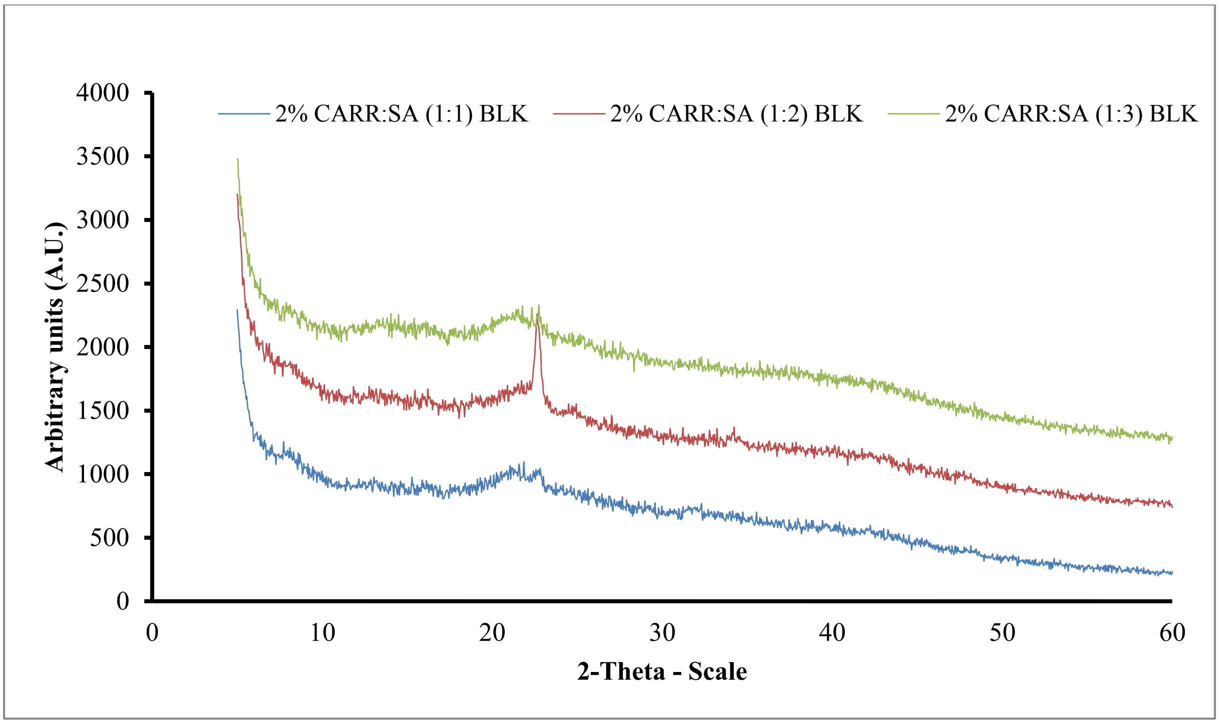

Figure S8. XRD diffractograms of 2% CARR:SA (1:1, 1:2 and 1:3) BLK.

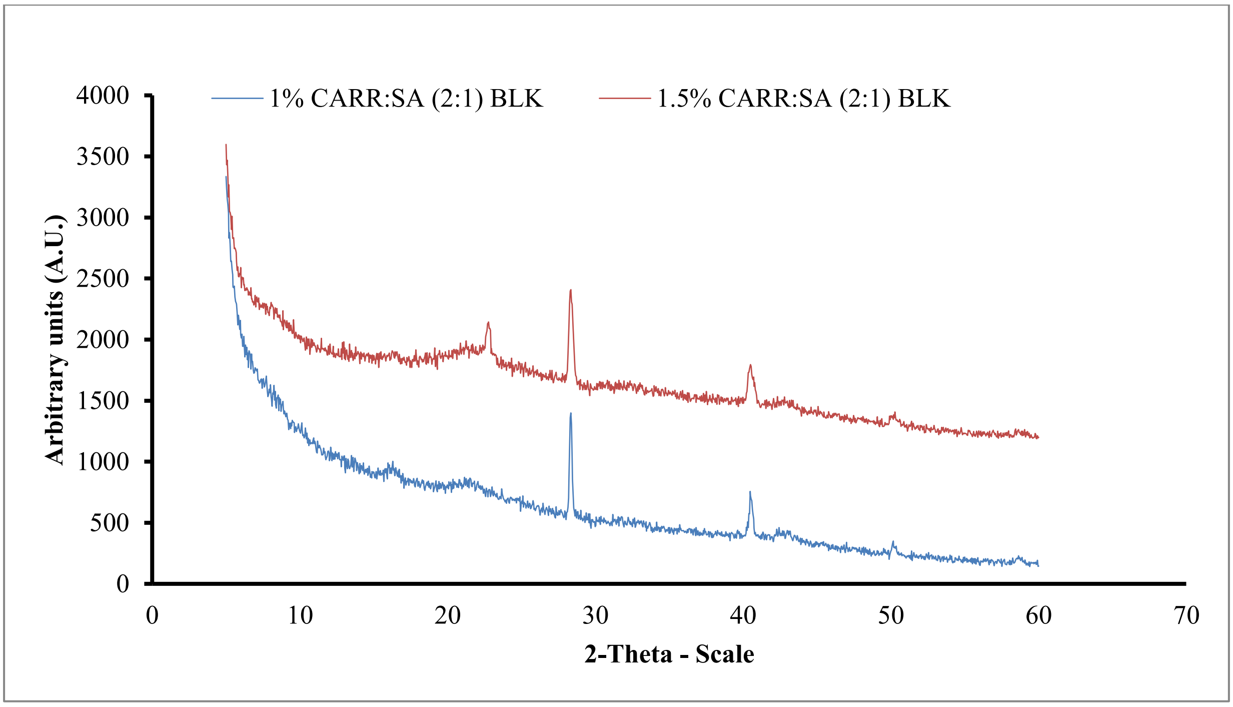

Figure S9. XRD diffractograms of 1% CARR:SA (2:1) BLK and 1.5% CARR:SA (2:1) BLK.

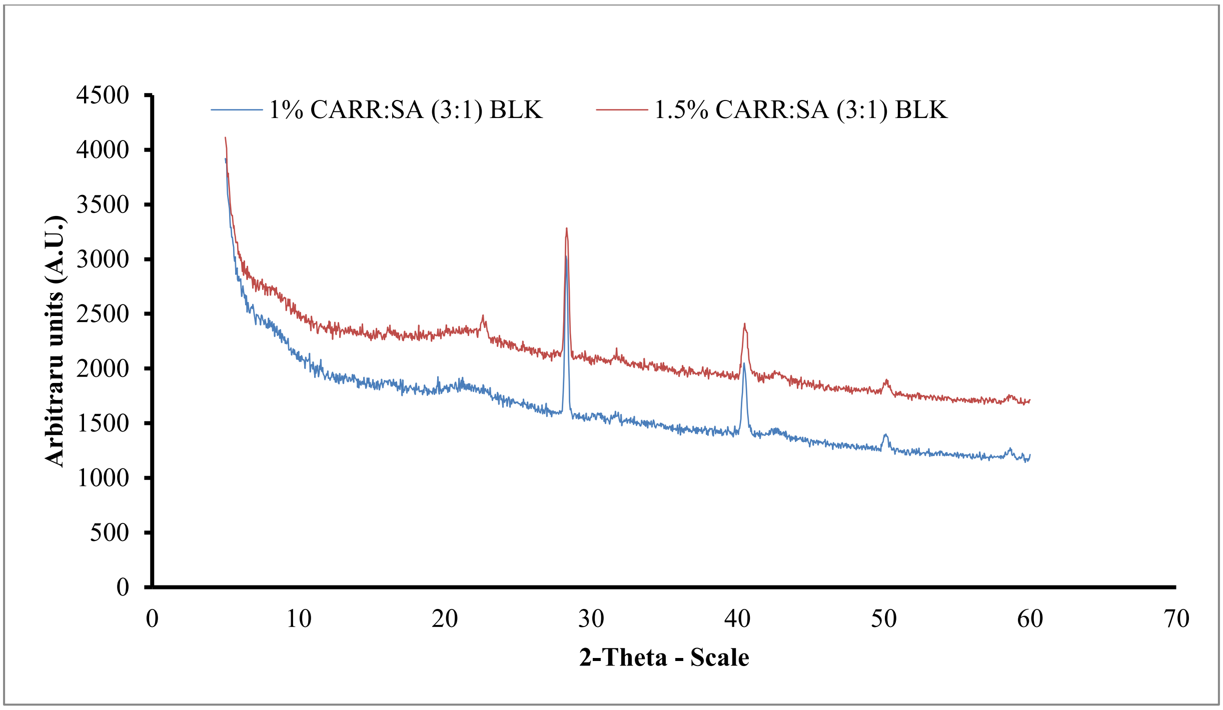

Figure S10. XRD diffractograms of 1% CARR:SA (3:1) BLK and 1.5% CARR:SA (3:1) BLK.

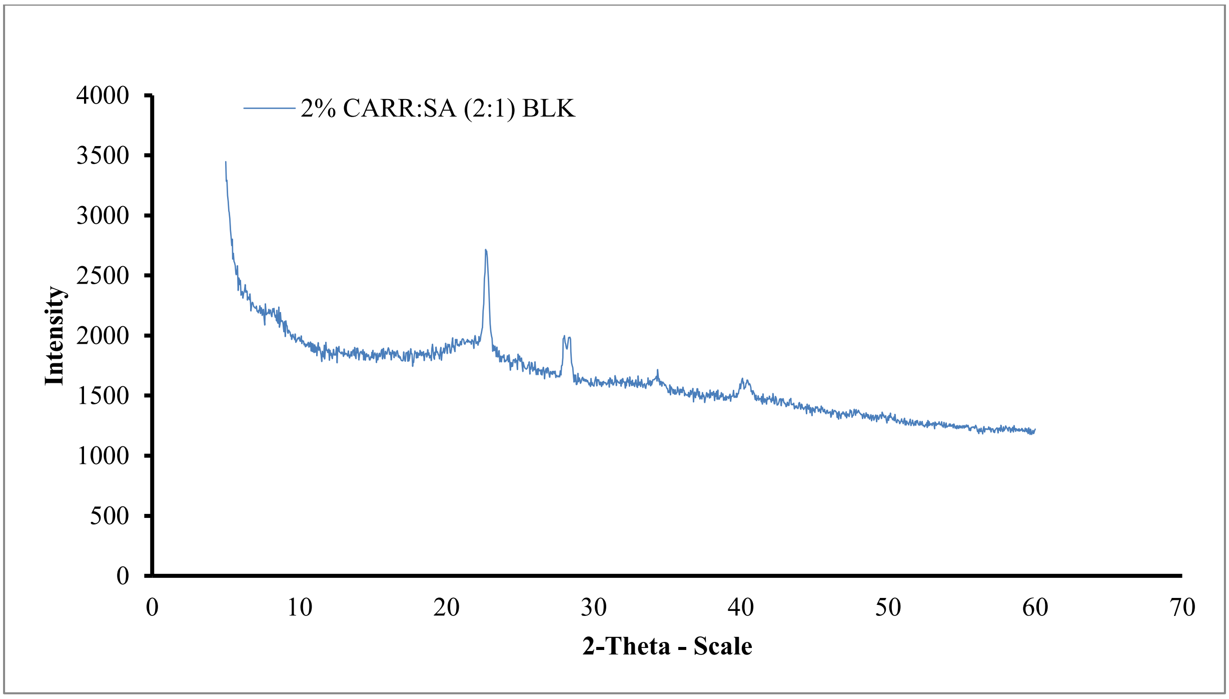

Figure S11. XRD diffractogram of 2% CARR:SA (2:1).
